# Metrnl/C‐KIT Axis Attenuates Early Brain Injury Following Subarachnoid Hemorrhage by Inhibiting Neuronal Ferroptosis

**DOI:** 10.1111/cns.70286

**Published:** 2025-02-21

**Authors:** You Zhou, Jiani Li, Ye Yuan, Hao Zhang, Xu Luo, Feng Wang, Yihao Tao, Jianhe Yue, Luyi Huang, Lei Wu, Yunxing Cao, Qian Yu, Qiuguang He

**Affiliations:** ^1^ Department of Critical Care Medicine, The Second Affiliated Hospital Chongqing Medical University Chongqing China; ^2^ Department of Neurology, The Second Affiliated Hospital Chongqing Medical University Chongqing China; ^3^ Department of Neurosurgery, The Second Affiliated Hospital Chongqing Medical University Chongqing China; ^4^ Key Laboratory of Molecular Biology for Diseases (Ministry of Education), Department of Infectious Diseases, Institute for Viral Hepatitis, The Second Affiliated Hospital Chongqing Medical University Chongqing China; ^5^ Department of Neurology Guangdong Second Provincial General Hospital Guangzhou Guangdong China; ^6^ Department of Neurosurgery, School of Medicine, The Second Affiliated Hospital Zhejiang University Hangzhou Zhejiang China

**Keywords:** C‐KIT, ferroptosis, Metrnl, subarachnoid hemorrhage

## Abstract

**Background and Purpose:**

Ferroptosis is a distinct form of cell death characterized by iron‐dependent lipid peroxidation and plays a crucial role in the early brain injury (EBI) following subarachnoid hemorrhage (SAH). As a newly discovered endogenous ligand for the C‐KIT receptor tyrosine kinase, meteorin‐like protein (Metrnl) exerts regulatory functions in oxidative stress and protects against various diseases. However, the specific role of the Metrnl/C‐KIT axis in neuronal ferroptosis during EBI following SAH remains to be elucidated.

**Methods:**

Sprague Dawley rats were used to establish the SAH model through endovascular perforation. r‐Metrnl was administered intranasally 1 h after SAH. Metrnl shRNA, C‐KIT inhibitor ISCK03, AMPK inhibitor dorsomorphin, and Nrf2 inhibitor ML385 were administered intracerebroventricularly or intraperitoneally before r‐Metrnl treatment to explore the underlying mechanisms. Neurobehavioral assessments, immunofluorescence, western blot, ELISA, Fluoro‐Jade C staining, transmission electron microscopy, and Nissl staining were conducted to evaluate the effects. Additionally, primary neuron culture with hemoglobin (Hb) stimulation was used for in vitro studies.

**Results:**

Phosphorylated C‐KIT and endogenous Metrnl levels were upregulated after SAH. Knockdown of Metrnl aggravated neurobehavioral deficits and neuronal ferroptosis, whereas r‐Metrnl treatment showed a protective effect. Mechanistically, r‐Metrnl significantly increased the protein levels of SLC7A11, GPX4, FTH, FSP1, and GSH, whereas it decreased the levels of ACSL4, 4HNE, and MDA in the ipsilateral hemisphere 24 h after SAH. Also, r‐Metrnl reduced mitochondrial shrinkage, increased mitochondrial crista, and decreased membrane density. However, the beneficial effects of r‐Metrnl were partially reversed by ISCK03, dorsomorphin, or ML385 treatment both in vivo and in vitro.

**Conclusions:**

Our study demonstrated that r‐Metrnl reduced neuronal ferroptosis and improved neurological outcomes after SAH by modulating the C‐KIT/AMPK/Nrf2 signaling pathway.

## Graphical abstract

The Metrnl‐attenuated neuronal ferroptosis via the activation of C‐KIT/AMPK/Nrf2 signaling pathway after SAH.

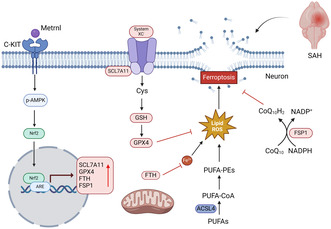

## 1 Introduction

Subarachnoid hemorrhage (SAH) is a critical health emergency with high rates of mortality and morbidity. This condition involves complex pathophysiological processes, including intracranial hypertension, cerebral blood flow reduction, initiation of cell death cascades, blood–brain barrier (BBB) disruption, and brain swelling [1]. Oxidative stress, a key factor in early brain injury (EBI) triggered by heme, induces neuronal apoptosis, ferroptosis, and tissue necrosis by overproducing reactive oxygen species (ROS) [2]. In addition, excessive ROS is most strongly linked to ferroptosis [2]. However, the precise mechanisms underlying ferroptosis in the context of SAH remain poorly characterized.

Ferroptosis is a distinct form of cell death caused by the loss of balance in cellular iron and redox status [3]. Ferroptosis is marked by increased lipid peroxidation and elevated intracellular ferrous iron (Fe^2+^) levels [4]. Following SAH, heme is released from lysed erythrocytes and is further metabolized into ferrous/ferric iron by microglia, accumulating in neurons through the transferrin (Tf)–Tf receptor system [5]. Lipid‐based ROS accumulates due to the heightened Fenton reaction triggered by excess iron, coupled with the depletion of intracellular glutathione (GSH) and glutathione peroxidase 4 (GPX4), and the disabling of cysteine/glutamate transporters (System Xc−) [6]. Studying the molecular mechanisms underlying ferroptosis, either by utilizing specific inhibitors, including liproxstatin‐1 and ferrostatin‐1, or by chelating iron, is key to formulating innovative and effective strategies against ferroptosis [3]. Recent studies suggested that ferroptosis contributes to EBI‐mediated neuronal damage [7]. However, the precise molecular mechanisms involved in ferroptosis are still not fully understood. Further exploration is required to identifying potential effective therapeutic targets for SAH treatment.

Meteroin‐like protein (Metrnl), also known as Meteorin‐β, Subfatin, Interleukin‐41, and Cometin, has recently been identified as a new neurotrophic factor that supports neurite outgrowth, cellular migration, and neuroprotection [8, 9]. It is extensively distributed across various tissues, including the liver, kidney, muscle, vessel, heart, brain, respiratory tract, and digestive tract [10]. It has been reported that Metrnl can alleviate oxidative stress, apoptosis, and cardiac dysfunction induced by doxorubicin in cardiac muscle cells [11]. Also, increasing evidence supports that Metrnl specifically binds to C‐KIT to initiate downstream signaling, which modulates several cellular processes such as survival, migration, and proliferation [12, 13, 14]. A recent study highlighted that Metrnl derived from myeloid cells could promote angiogenesis by binding to the C‐KIT receptor tyrosine kinase following myocardial infarction [15]. Furthermore, overexpression of Metrnl has been proved to significantly reduce LPS‐induced ferroptosis and subsequently decrease acute lung injury severity [16]. These evidence suggested that interaction between Metrnl and C‐KIT may also play an important role in regulating ferroptosis.

Studies showed that adenosine monophosphate–activated protein kinase (AMPK) activation is a key mechanism through which Metrnl elicits its biological functions [17, 18, 19, 20]. AMPK is an important metabolic sensor that regulates energy metabolism and mitochondrial functionality [20]. Nuclear factor erythroid 2–related factor 2 (Nrf2) is a key downstream effector of AMPK that controls the expression of several genes pivotal to anti‐ferroptosis, such as cystine/glutamate antiporter solute carrier family 7 member 11 (SLC7A11), GPX4, ferritin heavy chain (FTH), and ferroptosis suppressor protein 1 (FSP1) [21, 22]. Additionally, it has been demonstrated that activation of AMPK can help reduce ferroptosis by promoting the activity of Nrf2 [23]. Therefore, there is a strong possibility that Metrnl can alleviate ferroptosis by interacting with C‐KIT, which further activates the AMPK/Nrf2 pathway.

Taken together, in the present study, we conducted in vivo and in vitro experiments to prove that Metrnl could alleviate EBI by activating C‐KIT to moderate lipid peroxidation and inhibit ferroptosis via AMPK/Nrf2 pathway. To explore our hypothesis, endogenous Metrnl knockdown, exogenous Metrnl administration, and detection of ferroptotic‐related changes were performed. Also, neurological function was evaluated in rats.

## 2 Materials and Methods

### 2.1 Animals

All animal experiments were carried out in accordance with the National Institutes of Health (NIH) Guide for the Care and Use of Laboratory Animals and were approved by the Institutional Animal Care and Use Committee at Chongqing Medical University. The study used adult male Sprague Dawley (SD) rats weighing 280–300 g. The rats were housed in standard laboratory conditions with a temperature maintained at 22°C and relative humidity of 40%–60%. A 12‐h light/dark cycle was provided. The animals were kept in ventilated cages with bedding material regularly replaced and with free access to standard rodent chow and water.

### 2.2 SAH Model and SAH Grading

After isoflurane anesthesia, the rats were intubated and placed on mechanical ventilation. A 4‐0 monofilament punctured the bifurcation where the anterior and middle cerebral arteries meet by inserting it through the left external carotid artery into the internal carotid artery. The same surgical procedure was performed in the sham group, but the arterial puncture was omitted. Vital signs such as respiration, heart rate, skin color, and pedal reflex were monitored every 5 min to ensure appropriate anesthesia depth and to minimize distress. All procedures were performed according to previously established methods [24].

The severity of SAH was evaluated in a blinded assessment by one independent investigator using a scoring system based on previous studies [25]. In summary, the basal cistern was divided into six regions, and each was assigned a score from 0 to 3 depending on the extent of blood clot formation in the subarachnoid space. The maximum score was 18, with 0 indicating no blood. Rats with scores below 9 were excluded to ensure consistency in SAH severity across all subjects included in further analyses.

In this study, 424 rats were used, of which 322 rats underwent SAH induction. The overall mortality rate for SAH rats was 16.8% (54 out of 322). None of the rats died in the sham or naïve groups. Additionally, 14 SAH rats were excluded from the analysis due to low SAH grading scores. SAH grades were not significantly disparate among each group of SAH, as shown in Table 1.

**TABLE 1:** Summary of animal numbers and mortality.

Groups	Mortality	Excluded
Experiment 1		
Sham	0.0% (0/8)	0
SAH (3 h, 6 h, 12 h, 24 h, 72 h)	17.9% (7/39)	2
Experiment 2		
Naïve	0.0% (0/6)	0
Naïve + scr shRNA	0.0% (0/6)	0
Naïve + Metrnl shRNA	0.0% (0/6)	0
Sham	0.0% (0/20)	0
SAH	20.0% (5/25)	1
SAH + scr shRNA	16.7% (4/24)	2
SAH + Metrnl shRNA	16.7% (4/24)	1
Experiment 3		
Sham	0.0% (0/20)	0
SAH + PBS	20.0% (5/25)	1
SAH + r‐Metrnl (1.33 μg/kg)	25.0% (2/8)	0
SAH + r‐Metrnl (4 μg/kg)	16.7% (4/24)	1
SAH + r‐Metrnl (12 μg/kg)	25.0% (2/8)	0
Experiment 4		
Sham	0.0% (0/6)	0
SAH + PBS	14.2% (1/7)	0
SAH + r‐Metrnl (4 μg/kg)	14.2% (1/7)	0
Experiment 5		
Sham	0.0% (0/16)	0
SAH + PBS	20.0% (4/20)	1
SAH + r‐Metrnl	15.7% (3/19)	0
SAH + r‐Metrnl + DMSO (i.c.v.)	15.7% (3/19)	1
SAH + r‐Metrnl + ISCK03	20.0% (4/20)	0
SAH + r‐Metrnl + dorsomorphin	15.7% (3/19)	2
SAH + r‐Metrnl + DMSO (i.p.)	15.7% (3/19)	1
SAH + r‐Metrnl + ML385	20.0% (4/20)	1
Total		
Sham	0.0% (0/70)	0
Naïve	0.0% (0/18)	0
SAH	16.8% (54/322)	14

### 2.3 Cell Culture

Primary neuron culture and hemoglobin (Hb) stimulation were used as an in vitro model. Primary neurons were extracted from P1 SD rat pups. Briefly, whole brain tissues were removed aseptically from the pups' heads, and cortex tissues were separated from the brains. After removing the meninges and blood vessels, cortex tissues were lysed with 0.25% trypsin for 10 min at 37°C. Then, the lysis was terminated, and the tissues were dispersed by a Pasteur pipette. Neurons were seeded on poly‐l‐lysine‐coated plates with a neurobasal medium (Gibco, MD, USA), which contained 2% B_27_ (Gibco) and 0.5 mM glutamine, and were cultured in an incubator at 37°C, with 5% CO_2_. The medium was half changed every 3 days. The cells were then subjected to the in vitro experiments after 7 days of culture.

For in vitro model, rat primary neurons were seeded onto six‐well plates. Once the cultures reached maturity, the medium was fully replaced with a fresh medium containing 25 μM Hb (9008‐02‐0, Sigma, USA) and incubated for another 24 h.

### 2.4 Drug Administration

#### 2.4.1 Animal Experiments

Treatments were administered through three routes: intranasal (i.n.), intracerebroventricular (i.c.v.), and intraperitoneal (i.p.). One hour after SAH induction, recombinant Metrnl (r‐Metrnl, CSB‐EP719323RA, CUSABIO, Carlsbad, USA), dissolved in PBS, was administered intranasally, as per previously described methods [26]. PBS was used as a vehicle at an equal volume. r‐Metrnl was delivered in three different dosages (1.33 μg/kg, 4 μg/kg, and 12 μg/kg), alternately in the left and right nostrils with 5 μL per nostril at 5‐min intervals [20]. For long‐term effect assessments, r‐Metrnl was also administered on Days 2 and 3 after SAH as per the study protocol.

The i.c.v. administration was carried out as previously detailed [27]. Following isoflurane anesthesia, rats were fixed in a stereotaxic apparatus. A 10‐μl Hamilton syringe was delicately inserted into the left lateral ventricle through a cranial burr hole, aligned 0.9 mm posterior and 1.5 mm lateral to the bregma, reaching 3.3 mm beneath the skull's horizontal plane. To prevent leakage, the infusion of shRNA or drugs was carefully controlled at a 1 μL/min rate. After infusion, the needle was maintained for 5 min before slow withdrawal. The burr hole was then efficiently sealed with bone wax, and the surgical incision was closed. The sequences Metrnl short hairpin RNA (shRNA) and scrambled (scr) shRNA (TL702232V, OriGene Technologies, MD, USA) are listed as follows: sh‐Metrnl: 5′‐CCAGAACAGCAAGTGTCAGTCATTCACCT‐3′; scr shRNA: 5′‐GCACTACCAGAGCTAACTCAGATAGTACT‐3′. The lentivirus titer of shRNA was 1.0 × 10^8^ TU/mL. A volume of 5 μL lentivirus for Metrnl shRNA or scr shRNA was administered via the i.c.v. at 2 weeks before SAH. Prior to induction, ISCK03 (a C‐KIT specific inhibitor, 2.5 mg/kg, HY‐101443, MedChemExpress, NJ, USA) and dorsomorphin (an AMPK specific inhibitor, 1 mg/kg, HY‐13418A, MedChemExpress, NJ, USA) were dissolved in dimethyl sulfoxide (DMSO) and administered via i.c.v. injection 1 h beforehand. Additionally, ML385 (an Nrf2 specific inhibitor, 30 mg/kg, 6887, Tocris, MN, USA), dissolved in DMSO, was injected i.p. 1 h before the procedure.

#### 2.4.2 Neuron Culture Experiment

r‐Metrnl (5 μM) was added 1 h after Hb stimulation [20]. C‐KIT inhibitor (ISCK03, 5 μM) or AMPK inhibitor (orsomorphin, 10 μM) was administered 1 h before Hb stimulation.

### 2.5 Experiment Design

**FIGURE 1:**
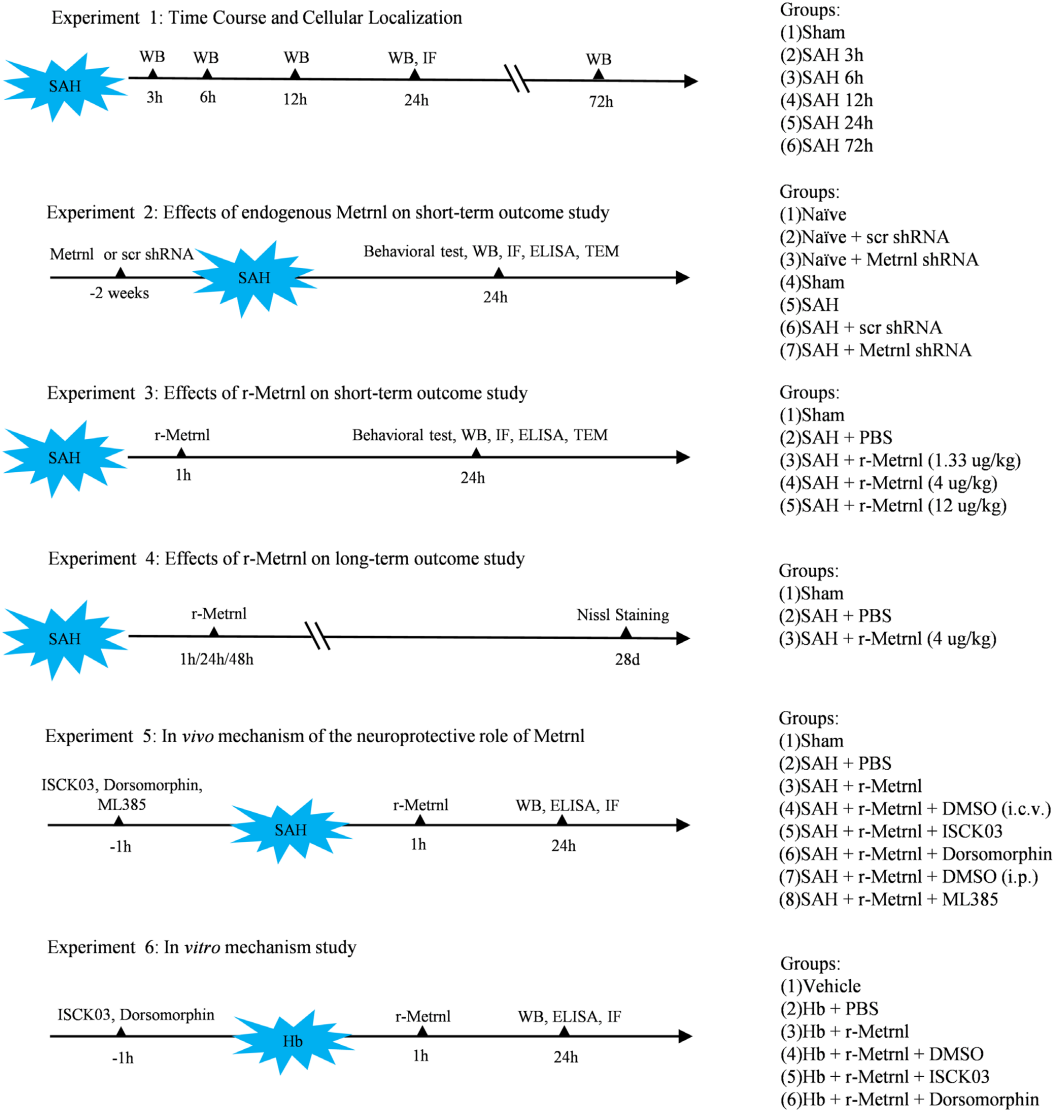
Experimental design.

The study was organized into six distinct sub‐experiments, with subjects randomly and blindly assigned to different groups (Figure 1). If any death or exclusion occurred, an additional animal was allocated as a replacement to ensure that each group maintained consistent numbers and the sample size was adequate for statistical analysis.

#### 2.5.1 Experiment 1: Time Course and Cell Localization of Endogenous Metrnl and p‐C‐KIT After SAH

For western blot analysis, 36 rats were randomly and evenly assigned to six groups: one sham group and groups at 3 h, 6 h, 12 h, 24 h, and 72 h after SAH. This experiment aimed to assess the expression of Metrnl, p‐C‐KIT, and C‐KIT. Additionally, to determine the spatial relationship between Metrnl and p‐C‐KIT with neurons, four rats were divided into sham (*n* = 2) and 24 h after SAH (*n* = 2) groups for double immunofluorescence analysis.

#### 2.5.2 Experiment 2: Role of Endogenous Metrnl in Short‐Term Outcomes and Neuronal Ferroptosis Following SAH in Rats

To explore the role of endogenous Metrnl in neuronal ferroptosis after SAH, Metrnl shRNA was used to assess both neuronal ferroptosis and neurobehavioral outcomes 24 h after SAH. Initially, 18 naïve rats were randomly allocated to the naïve, scr shRNA, or Metrnl shRNA group (*n* = 6/group). Both shRNA types were administered via i.c.v. injection 2 weeks prior to brain tissue collection to evaluate the knockdown efficiency.

Another set of 80 rats was randomly divided into four groups (*n* = 20/group): Sham, SAH, SAH + scr shRNA, and SAH + Metrnl shRNA. Neurobehavioral tests (*n* = 6/group) were performed. Western blot (*n* = 6/group) was used to assess protein levels of Metrnl, SLC7A11, GPX4, FSP1, FTH, and acyl‐CoA synthetase long‐chain family member 4 (ACSL4). The levels of malondialdehyde (MDA) and GSH were assessed using MDA and GSH assay kits. Immunofluorescence staining of 4HNE, GPX4, and Fluoro‐Jade C (FJC) (*n* = 4/group) was performed. Transmission electron microscopy (TEM) was used to evaluate the morphological changes of mitochondria.

#### 2.5.3 Experiment 3: Effect of Exogenous Metrnl Treatment on Short‐Term Outcomes and Neuronal Ferroptosis 24 h After SAH in Rats

To determine the best treatment dosage for r‐Metrnl, 30 rats were randomly assigned to five groups for neurobehavior evaluation (*n* = 6/group): sham, SAH + PBS, SAH + r‐Metrnl (1.33 μg/kg), SAH + r‐Metrnl (4 μg/kg), and SAH + r‐Metrnl (12 μg/kg). r‐Metrnl was administered i.n. at 1 h following SAH. Neurobehavioral tests were conducted at 24 h after SAH. Based on the results of the neurological tests, an additional 42 rats were randomly assigned to sham, SAH + PBS, and SAH + r‐Metrnl (best dose) groups to observe the effect of r‐Metrnl on neuronal ferroptosis. Western blot analysis (*n* = 6/group) assessed Metrnl, SLC7A11, GPX4, FSP1, FTH, and ACSL4 protein levels. Levels of MDA and GSH were measured using assay kits (*n* = 6/group). Immunofluorescence staining for 4NHE, GPX4, and FJC was performed (*n* = 4/group). TEM was used to evaluate morphological changes in mitochondria.

#### 2.5.4 Experiment 4: Effect of Exogenous r‐Metrnl Treatment on Long‐Term Neuronal Degeneration Following SAH in Rats

To determine the efficacy of exogenous r‐Metrnl in mitigating long‐term neuronal degeneration after SAH, rats were divided into three groups (*n* = 6/group) for analysis at 28 days after SAH: Sham, SAH + PBS, and SAH + r‐Metrnl. Neuronal degeneration within the ipsilateral hippocampus was examined using Nissl staining.

#### 2.5.5 Experiment 5: In Vivo Mechanism Study (Metrnl/C‐KIT/AMPK/Nrf2 Signaling Pathway)

To investigate the potential molecular mechanism of C‐KIT activation, 96 rats were randomly divided into six groups (*n* = 16/group): Sham, SAH + PBS, SAH + r‐Metrnl, SAH + r‐Metrnl + DMSO (i.c.v.), SAH + r‐Metrnl + ISCK03 (C‐KIT inhibitor), and SAH + r‐Metrnl + dorsomorphin (AMPK inhibitor). To further explore the Nrf2 signaling pathway in the Metrnl‐mediated anti‐ferroptosis effect after SAH, an additional 32 rats were randomly assigned to two groups (*n* = 16/group): SAH + r‐Metrnl + DMSO (i.p.) and SAH + r‐Metrnl + ML385 (Nrf2 inhibitor). r‐Metrnl or PBS was administered via the i.n. route at 1 h after SAH. ISCK03, dorsomorphin, or DMSO was administered via i.c.v. injection 1 h before SAH. ML385 or DMSO was administered via i.p. injection 1 h before SAH. Western blot was used to assess the expression levels of the Metrnl/C‐KIT/AMPK/Nrf2 signaling axis (p‐C‐KIT, C‐KIT, p‐AMPK, AMPK, Nrf2), ferroptosis markers (SLC7A11, GPX4, FSP1, FTH, ACSL4) (*n* = 6/group). ELISA was used to evaluate MDA and GSH levels (*n* = 6/group). Immunofluorescence staining for 4NHE was performed (*n* = 4/group).

#### 2.5.6 Experiment 6: In Vitro Mechanism Study

Cultured cells were randomly divided into six groups: Sham, Hb + PBS, Hb + r‐Metrnl, Hb + r‐Metrnl + DMSO, Hb + r‐Metrnl + ISCK03 (C‐KIT inhibitor), and Hb + r‐Metrnl + dorsomorphin (AMPK inhibitor). Western blot was performed to measure the expression of p‐C‐KIT, C‐KIT, p‐AMPK, AMPK, Nrf2, SLC7A11, GPX4, FSP1, FTH, and ACSL4 levels (*n* = 6/group). ELISA was performed to measure MDA and GSH levels (*n* = 6/group). Mitochondrial membrane potential was assessed with the TMRE kit (*n* = 6/group).

### 2.6 Neurobehavioral Test

Short‐term neurological function was evaluated using two established tests: the modified Garcia test, scoring from 0 to 18, and the beam balance test, scoring from 0 to 4. The Garcia test measures response, alertness, coordination, and motor abilities, whereas the beam balance test focuses on complex movements and coordination [28]. Higher scores indicate superior performance. All the tests were performed by one independent investigator who was blinded to the group assignments and interventions.

### 2.7 Immunofluorescence Staining

Following established protocols, double immunofluorescence staining was performed [29]. Brain tissues were initially fixed in formalin for 24 h and then dehydrated through a sucrose gradient. Samples were embedded in OCT and sectioned into 10‐μm‐thick coronal slices. After rinsing in 0.01 M PBS, sections were blocked with 5% donkey serum at room temperature for 2 h before an overnight incubation at 4°C with primary antibodies: anti‐Metrnl (1:50, orb185592, Biorbyt, MA, UK), anti‐p‐C‐KIT (1:50, BS‐12916R, Thermo Fisher Scientific, Carlsbad, USA), anti‐4HNE (1:50, MA5‐27570, Thermo Fisher Scientific, Carlsbad, USA), anti‐GPX4 (1:100, ab125066, Abcam, MA, USA), and anti‐NeuN (1:100, ab177487, Abcam, MA, USA). The next day, slices were incubated with secondary fluorophore‐conjugated antibodies (1:500, Jackson ImmunoResearch) for 1 h. After incubation, a fluorescence microscope was utilized to visualize and photograph the slices. 4HNE‐ or GPX4‐positive cells were identified and quantified at 200× magnification. For each group containing four rats, the average cell numbers were calculated from four slices per rat.

### 2.8 FJC Staining

We used FJC staining with a ready‐to‐dilute kit (TR‐100‐FJ, Biosensis, USA) to identify degenerating neurons in the basal temporal cortex, adhering to the prior protocol [30]. FJC‐positive cells in the ipsilateral temporal cortex were identified and quantified using a fluorescence microscope at 200× magnification. For each group containing four rats, the average cell numbers were calculated from four slices per rat.

### 2.9 Mitochondrial Membrane Potential Analysis

The mitochondrial membrane potential of primary neurons was measured using the TMRE kit (T669, Thermo Fisher Scientific, Carlsbad, USA) according to the manufacturer's instructions. Briefly, primary neurons were incubated with the TMRE working solution for 30 min at 37°C. After incubation, the cells were washed three times with serum‐free DMEM to remove excess dye. The cells were observed and photographed using a fluorescence microscope at 400× magnification. The mean fluorescence intensity was analyzed by ImageJ software. The average intensity was calculated from six slides per group.

### 2.10 Nissl Staining

Nissl staining was conducted to examine neuronal damage in the ipsilateral hippocampus on the 28th day after SAH. A 0.5% crystal violet solution was used for staining, following established protocols [31]. The slices were observed and photographed at 200× magnification using a light microscope. Surviving neurons were characterized by pale nuclei, large cell bodies, and abundant cytoplasm. In contrast, neurons with shrunken cytoplasm and condensed staining were considered injured [31]. In the long‐term study, for each group containing six rats, the average surviving neurons in hippocampal areas, including cornu ammonis (CA)1, CA3, and the dentate gyrus (DG), were quantified from four slices per rat.

### 2.11 ELISA Assay

Lipid peroxidation was assessed using the MDA Assay Kit (MBS162002, MyBioSource, CA, USA), and GSH levels were determined using the GSH Assay Kit (Beyotime, Shanghai, China), both in accordance with the manufacturer's protocols.

### 2.12 Western Blot Analysis

Western blot was conducted as in the previous studies [25, 27]. Brain tissues or primary neurons underwent lysis in RIPA buffer to isolate proteins. In all, 10% of SDS‐PAGE gels were used for electrophoresis and proteins were then transferred to PVDF membranes (Millipore, USA). The membranes were treated with 5% skimmed milk at room temperature for 60 min and then incubated with primary antibodies at 4°C overnight. The primary antibodies used are listed as follows: anti‐Metrnl (1:500, ab235775, Abcam, MA, USA), anti‐p‐C‐KIT (1:500, GTX25616, GeneTex, CA, USA), anti‐C‐KIT (1:500, ab256345, Abcam, MA, USA), anti‐p‐AMPK (1:500, 2535, Cell Signaling Technology, MA, USA), anti‐AMPK (1:500, 2532, Cell Signaling Technology, MA, USA), anti‐Nrf2 (1:1000, ab137550, Abcam, MA, USA), anti‐SLC7A11 (1:1000, ab175186, Abcam, MA, USA), anti‐GPX4 (1:1000, ab125066, Abcam, MA, USA), anti‐FSP1 (1:1000, sc‐377,120, Santa Cruz Biotechnology, TX, USA), anti‐FTH (1:1000, sc‐376,594, Santa Cruz Biotechnology, TX, USA), anti‐ACSL4 (1:1000, MA5‐42523, Thermo Fisher Scientific, CA, USA), and anti‐β‐actin (1:3000, Santa Cruz Biotechnology, TX, USA). The following day, the membrane was treated with horseradish peroxidase (HRP)–linked secondary antibodies for 60 min at room temperature. The detection of immunoreactive signals was achieved using enhanced chemiluminescence reagents (Millipore, USA). Subsequently, the signals were quantified with Image J software, with β‐actin used as a reference for normalization.

### 2.13 Transmission Electron Microscopy

Ipsilateral basal temporal cortex tissue was fixed for 2 h in an electron microscopy fixative solution containing 2.5% glutaraldehyde. Following dehydration, embedding, and sectioning at 100 nm, the samples were stained with 4% uranyl acetate for 20 min and 0.5% lead citrate for 5 min. The sections were then examined using a TEM (Hitachi H 7500, Japan). At a magnification of 3000×, the assessment of damaged neuronal mitochondria involved observing shrunken structures, vanished cristae, and denser membranes. For each group containing four rats, the average percentage of impaired neuronal mitochondria was counted in four sections per rat.

### 2.14 Statistical Analysis

Statistical assessments were performed using GraphPad Prism 8 software (San Diego, CA, USA). Protein levels, immunostaining, ELISA, Nissl staining, TEM, and neurological scores underwent the Shapiro–Wilk test for normal distribution and Levene's test for variance consistency. Subsequent statistical processing was conducted using one‐way ANOVA and Tukey's post hoc test. For data that did not conform to a normal distribution or exhibited nonhomogeneous variance, the Mann–Whitney *U* test or Kruskal–Wallis test was applied. The results are presented as mean ± SD, with a *p*‐value < 0.05 indicating statistical significance.

## 3 Results

### 3.1 Time Course of Endogenous Protein Levels and Cellular Localization of Metrnl and p‐C‐KIT in Ipsilateral Hemisphere After SAH

**FIGURE 2:**
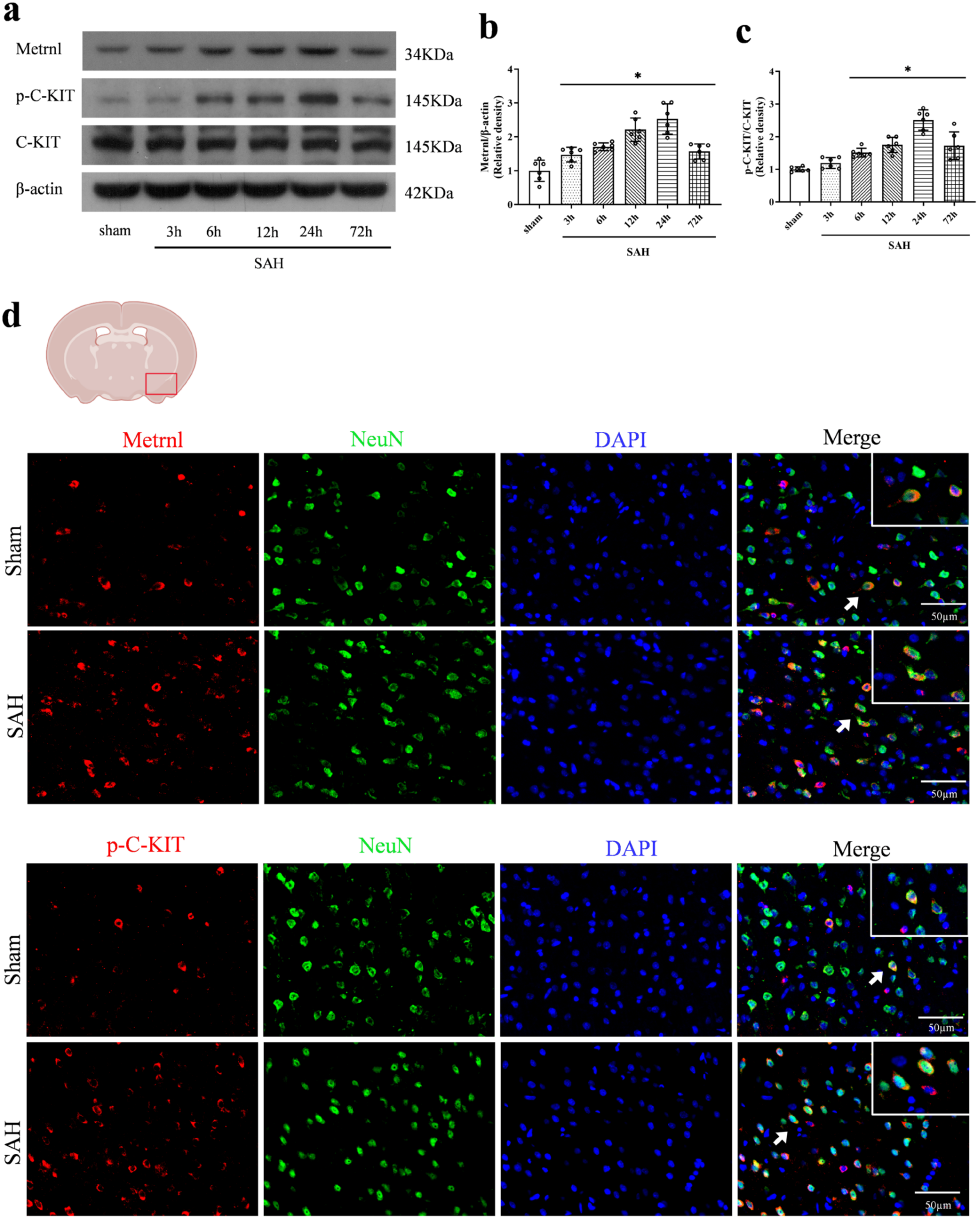
Time course of Metrnl and p‐C‐KIT and cellular locations of Metrnl and p‐C‐KIT after SAH. (a, c) Representative immunoblots bands and quantitative analysis of Metrnl, p‐C‐KIT, and C‐KIT expression. Data were represented as mean ± SD. *n* = 6 per group. *p < 0.05 vs. Sham group. (d) Representative double immunofluorescence staining microphotograph of Metrnl and p‐C‐KIT (red) with neuronal nuclei (NeuN, green) at 24 h after SAH. Scale bar = 50 μm, *n* = 2 per group.

The levels of endogenous Metrnl and p‐C‐KIT in the left cerebral hemisphere were measured using Western blot. Compared to the sham group, Metrnl levels significantly increased 6 h and peaked at 24 h after SAH (*p* < 0.05, Figure 2a,b). Similarly, p‐C‐KIT levels showed significant upregulation, which peaked at 24 h after SAH (*p* < 0.05, Figure 2a,c). Furthermore, immunofluorescence staining demonstrated that Metrnl and p‐C‐KIT co‐localized with neurons in the cortex of the left hemisphere (Figure 2d).

### 3.2 Endogenous Brain Metrnl Knockdown Exacerbated Neurological Deficits and Neuronal Degeneration After SAH

**FIGURE 3:**
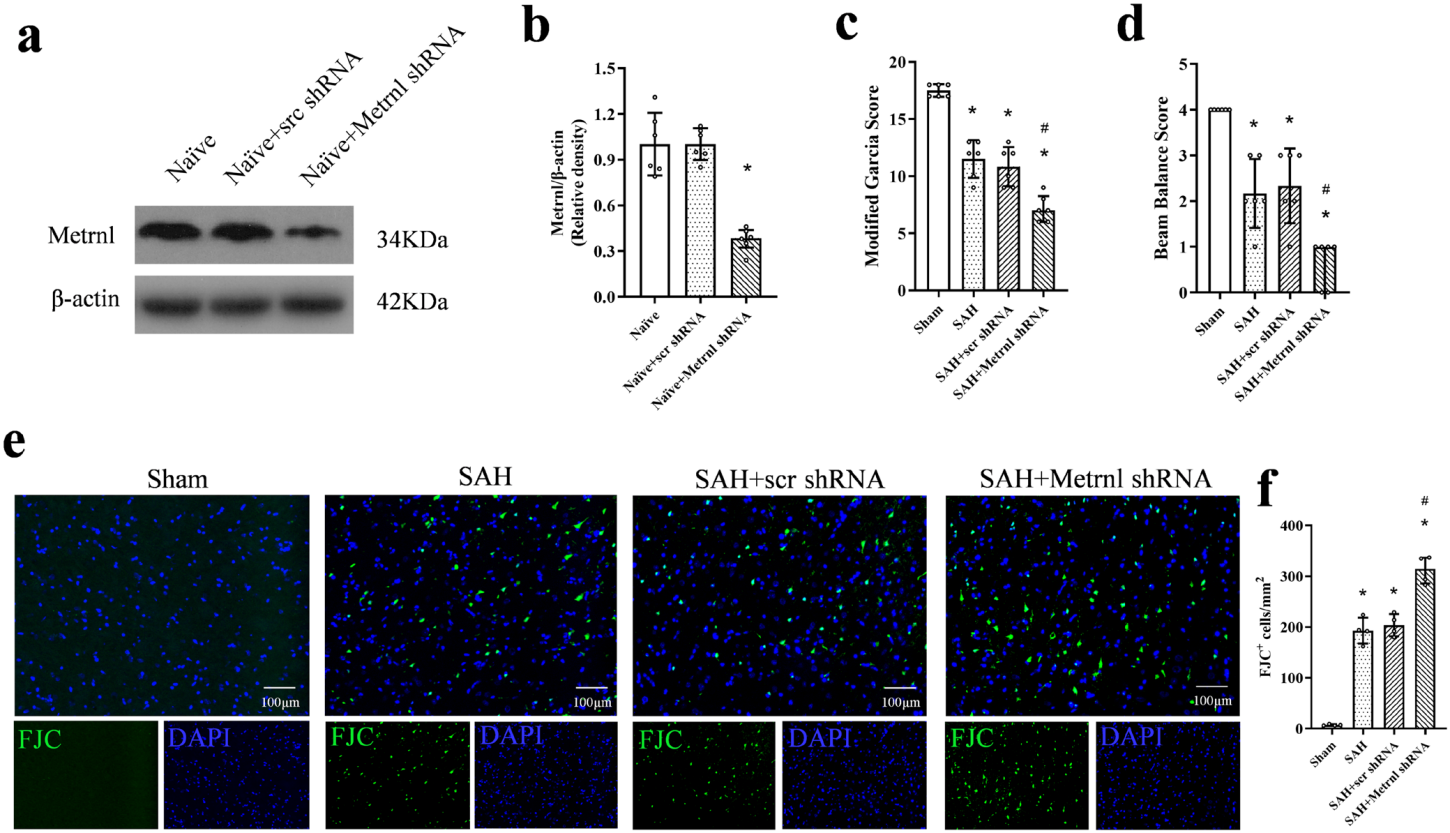
Endogenous brain Metrnl knockdown exacerbated neurological deficits and neuronal degeneration after SAH. (a, b) Representative western blot bands and densitometric quantification of Metrnl demonstrated the knockdown efficacy of Metrnl shRNA on brain Metrnl protein levels in naïve rats. *n* = 6 per group. *p < 0.05 vs. naïve + scr shRNA. (c, d) Rat performance evaluated by modified Garcia scores and beam balance scores at 24 h after SAH. *n* = 6 per group. (e, f) Representative image and quantitative analyses of FJC at 24 h after SAH. Scale bar = 100 μm, *n* = 4 per group. Data were represented as mean ± SD. *p < 0.05 vs. Sham; ^#^ *p* < 0.05 vs. SAH + scr shRNA.

To investigate the role of endogenous Metrnl in ferroptosis following SAH, Metrnl shRNA was administered 2 weeks before SAH induction. Western blot analysis confirmed a significant decrease in Metrnl protein levels after Metrnl knockdown (*p* < 0.05, Figure 3a,b). Neurobehavioral assessments, including modified Garcia and beam balance tests, revealed significantly lower scores in SAH rats compared to the sham group (*p* < 0.05, Figure 3c,d). Moreover, Metrnl knockdown exacerbated neurobehavioral deficits relative to the SAH group or the SAH + scr shRNA group (*p* < 0.05, Figure 3c,d).

FJC staining revealed that SAH led to neuronal degeneration. Metrnl knockdown further worsened neuronal degeneration compared to the SAH group or the SAH + scr shRNA group (*p* < 0.05, Figure 3e,f).

### 3.3 Endogenous Brain Metrnl Knockdown Exacerbated Neuronal Ferroptosis After SAH

**FIGURE 4:**
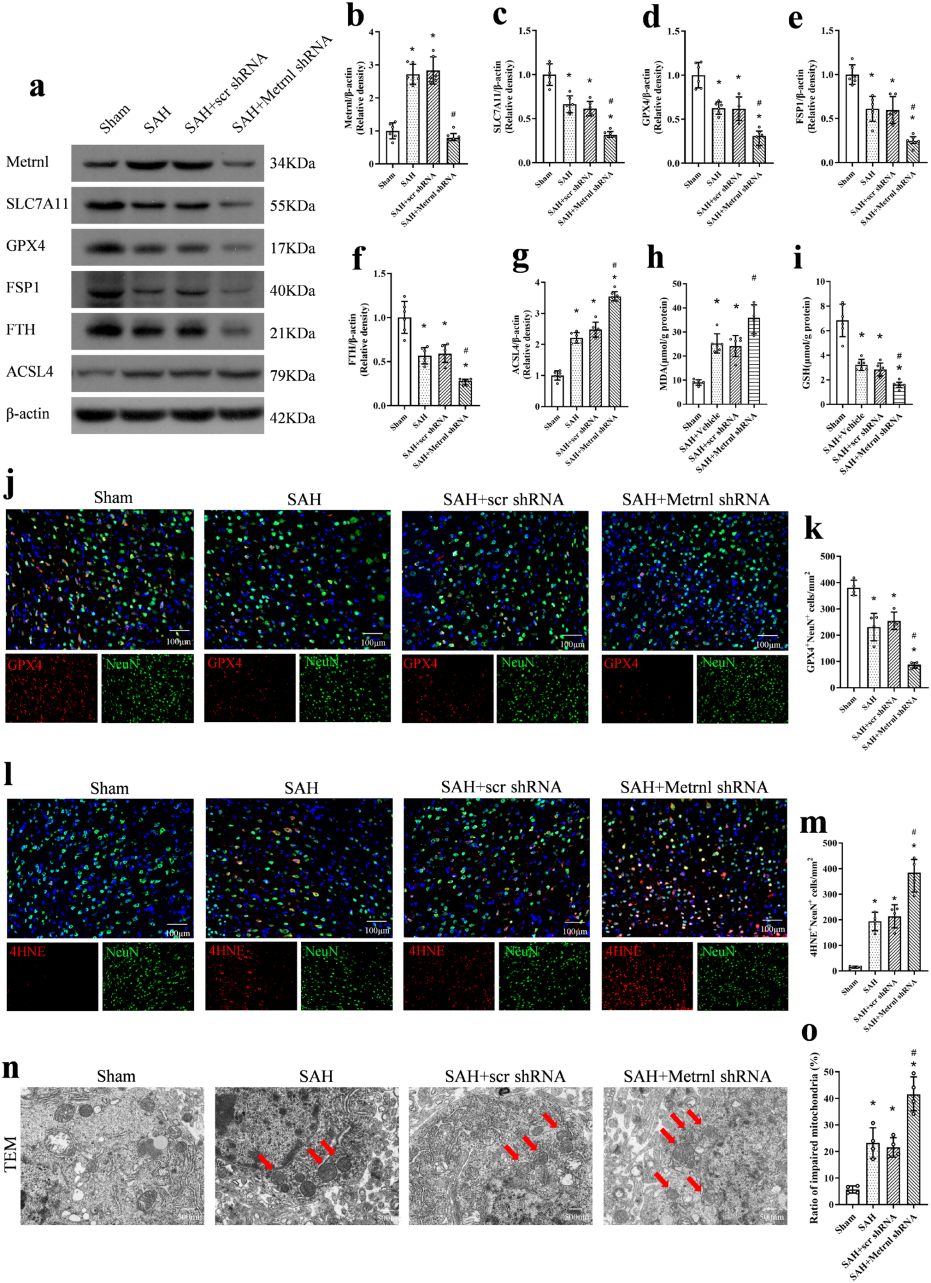
Endogenous brain Metrnl knockdown exacerbated neuronal ferroptosis after SAH. (a–g) Representative western blot bands and densitometric quantification of Metrnl, SLC7A11, GPX4, FSP1, FTH, and ACSL4. *n* = 6 per group. (h, i) The MDA and GSH levels. *n* = 6 per group. (j–m) Representative image and quantitative analyses of GPX4 and 4HNE at 24 h after SAH, scale bar = 100 μm, *n* = 4 per group. (n, o) Representative images and quantitative analysis of impaired mitochondria observed by transmission electron microscope in each group. Red arrows indicate impaired mitochondria in neurons. Scale bar = 500 nm, *n* = 4 per group. Data were represented as mean ± SD. *p < 0.05 vs. Sham; ^#^ *p* < 0.05 vs. SAH + scr shRNA.

Metrnl protein levels at 24 h after SAH were markedly lower in the SAH + Metrnl shRNA group compared to the SAH group and the SAH + scr shRNA group (*p* < 0.05, Figure 4a,b). Compared to the sham group, SAH rats showed significantly higher expressions of ACSL4 and MDA but lower levels of SLC7A11, GPX4, FSP1, FTH, and GSH at 24 h after SAH (*p* < 0.05, Figure 4c–g). When comparing the SAH group to the SAH + scr shRNA group, there was no significant difference observed in the protein levels of Metrnl and ferroptosis indicators (*p* > 0.05, Figure 4a–i), including SLC7A11, GPX4, FSP1, FTH, ACSL4, MDA, and GSH. Metrnl knockdown significantly exacerbated the SAH‐induced increase in ACSL4 and MDA, and further decreased the expression of SLC7A11, GPX4, FSP1, FTH, and GSH compared to the SAH group or the SAH + scr shRNA group (*p* < 0.05, Figure 4a–i).

To explore the specific location where ferroptosis happened, we performed immunofluorescence experiments to co‐stain neurons with GPX4 and 4HNE. Our findings indicated that the knockdown of Metrnl decreased GPX4‐positive neurons in the ipsilateral cortex at 24 h after SAH, compared to the SAH + scr shRNA group (*p* < 0.05, Figure 4j,k). In addition, the level of 4HNE was increased in the ipsilateral cortex after SAH. Consistently, Metrnl knockdown led to an increase in the number of 4HNE‐positive neurons, compared to both the SAH group and the SAH + scr shRNA group (*p* < 0.05, Figure 4l,m).

Mitochondria in ferroptosis can often exhibit morphological changes such as condensation and shrinkage, a reduction or disappearance of cristae, and outer membrane damage. In our study, all these characteristics could be observed in the SAH group under TEM, which were further intensified by Metrnl knockdown (*p* < 0.05, Figure 4n,o).

### 3.4 Exogenous r‐Metrnl Treatment Attenuated Neurological Deficit and Neuronal Degeneration After SAH

**FIGURE 5:**
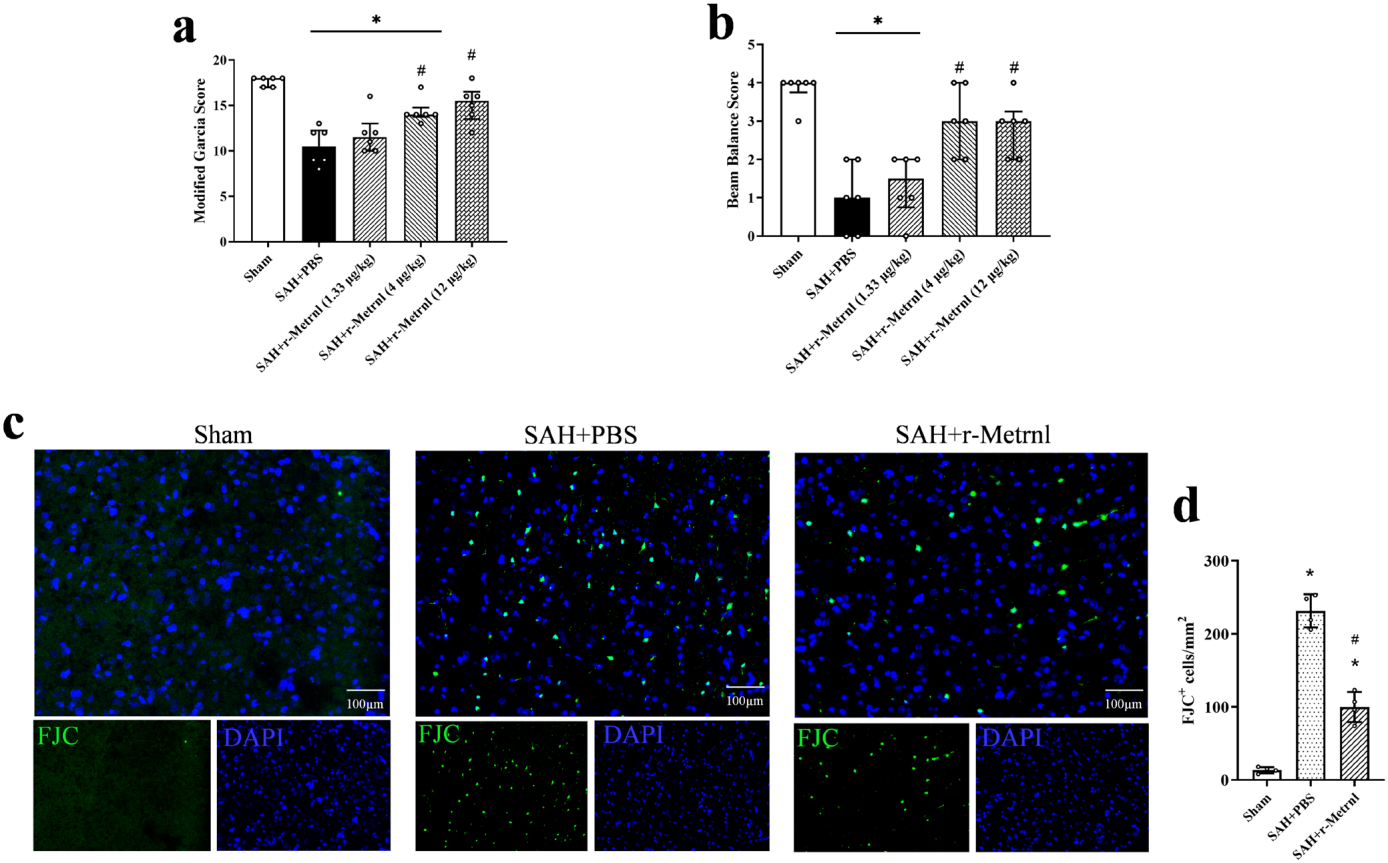
Exogenous r‐Metrnl treatment attenuated neurological deficits and neuronal degeneration after SAH. (a, b) Rat performance evaluated by modified Garcia scores and beam balance scores at 24 h after SAH. *n* = 6 per group. (c, d) Representative image and quantitative analyses of FJC at 24 h after SAH. Scale bar = 100 μm, *n* = 4 per group. Data were represented as mean ± SD. *p < 0.05 vs. Sham; ^#^ *p* < 0.05 vs. SAH + PBS.

After SAH induction, marked neurological impairments were displayed compared to the Sham group. Intranasal administration of r‐Metrnl (4 μg/kg and 12 μg/kg) significantly improved neurological function 24 h after SAH compared to the SAH + PBS group (*p* < 0.05, Figure 5a,b). Based on the neurobehavioral assessments, the medium dosage of r‐Metrnl (4 μg/kg) was selected for further experiments. FJC staining revealed that SAH‐induced neuronal damage was significantly mitigated by r‐Metrnl treatment (*p* < 0.05, Figure 5c,d).

### 3.5 Exogenous r‐Metrnl Treatment Attenuated Neuronal Ferroptosis After SAH

**FIGURE 6:**
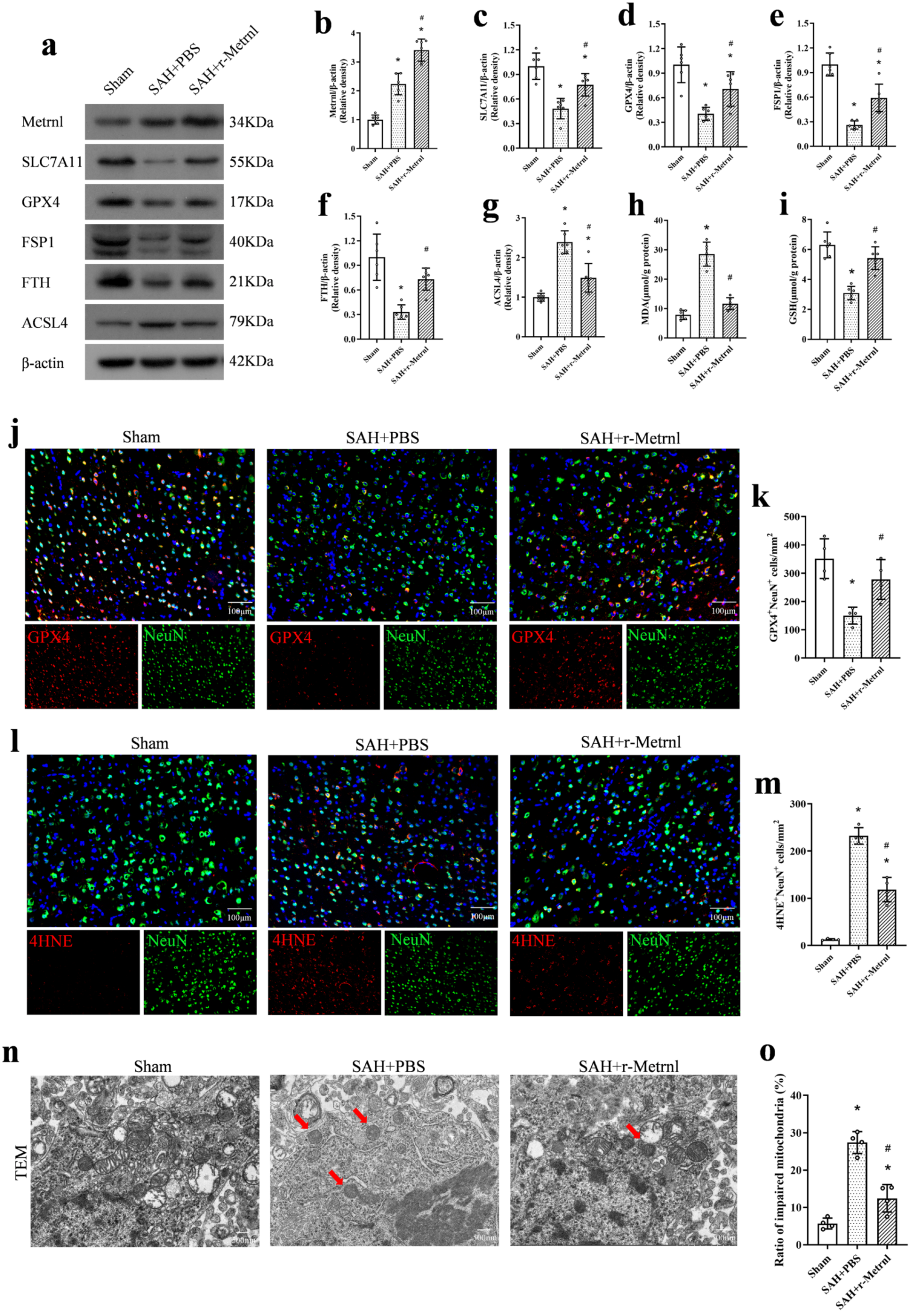
Exogenous r‐Metrnl treatment attenuated neuronal ferroptosis after SAH. (a–g) Representative western blot bands and densitometric quantification of Metrnl, SLC7A11, GPX4, FSP1, FTH, and ACSL4. *n* = 6 per group. (h, i) The MDA and GSH levels. *n* = 6 per group. (j–m) Representative images and quantitative analyses of GPX4 and 4HNE at 24 h after SAH. Scale bar = 100 μm, *n* = 4 per group. (n, o) Representative images and quantitative analysis of impaired mitochondria observed by transmission electron microscope in each group. Red arrows indicate impaired mitochondria in neurons. Scale bar = 500 nm, *n* = 4 per group. Data were represented as mean ± SD. *p < 0.05 vs. Sham; ^#^ *p* < 0.05 vs. SAH + PBS.

Western blot analysis revealed that compared to the SAH + PBS group, r‐Metrnl treatment significantly decreased the level of the pro‐ferroptosis protein ACSL4 and increased the expression of SLC7A11, GPX4, FSP1, and FTH at 24 h after SAH (*p* < 0.05, Figure 6a–g). Moreover, ELISA results demonstrated that r‐Metrnl treatment significantly improved GSH levels and reduced MDA expression compared to the SAH + PBS group (*p* < 0.05, Figure 6h,i).

Immunofluorescence analysis of GPX4 in the ipsilateral cortex indicated that SAH induction significantly reduced GPX4‐positive neurons in the SAH group compared to the Sham group, and r‐Metrnl restored the expression of GPX4 (*p* < 0.05, Figure 6j,k). Furthermore, r‐Metrnl treatment significantly decreased the number of 4HNE‐positive neurons 24 h after SAH compared to the untreated SAH group (*p* < 0.05, Figure 6l,m). Intranasal r‐Metrnl treatment alleviated the morphological changes of SAH‐induced mitochondrial atrophy, including reduced mitochondrial membrane density and cristae loss, as observed under TEM (*p* < 0.05, Figure 6n,o).

### 3.6 Intranasal r‐Metrnl Decreased Neuronal Degeneration in the Hippocampus Region on 28 Days After SAH

**FIGURE 7:**
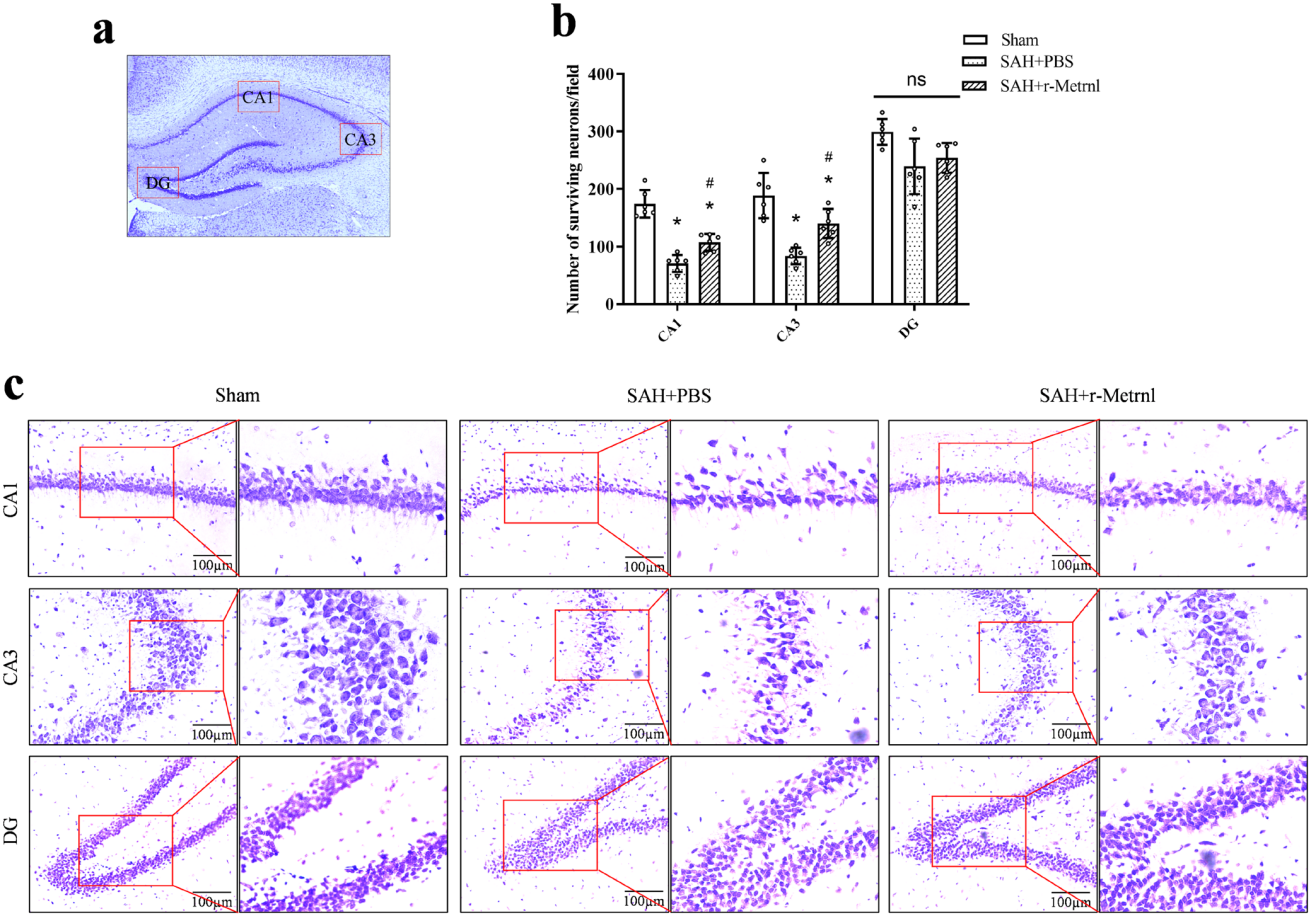
r‐Metrnl administration decreased neuronal degeneration in the hippocampus region 28 days after SAH. (a) Representative microphotograph of Nissl staining showed the location of CA1, CA3, and DG regions within the left hippocampus. (b, c) Representative microphotographs of Nissl staining quantifications of the surviving neurons in CA1, CA3, and DG hippocampal regions. Scale bar = 100 μm, *n* = 6 per group. Data are represented as mean ± SD. *p < 0.05 vs. Sham group; ^#^ *p* < 0.05 vs. SAH + PBS group.

Nissl staining performed 28 days after SAH revealed increased neuronal loss and morphological shrinkage within the CA1 and CA3 regions of the ipsilateral hippocampus in the SAH + PBS group compared to the sham group (*p* < 0.05, Figure 7a–c). In contrast, the SAH + r‐Metrnl group exhibited reduced neuronal degeneration, underscoring the neuroprotective impact of r‐Metrnl treatment (*p* < 0.05, Figure 7a–c).

### 3.7 R‐Metrnl Attenuated Neuronal Ferroptosis via C‐KIT/AMPK/Nrf2 Signaling Pathway After SAH

**FIGURE 8:**
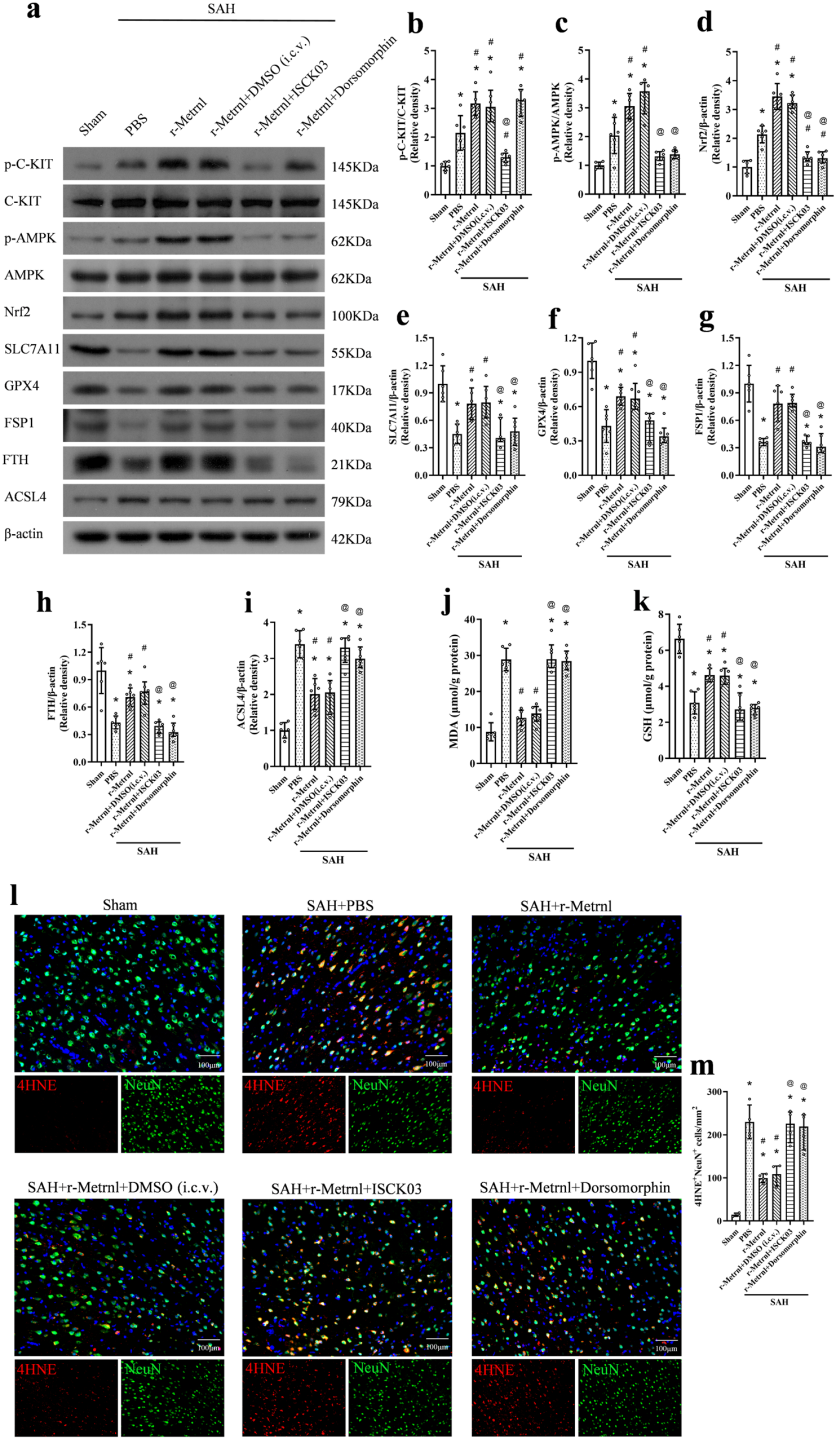
Inhibition of C‐KIT or AMPK reversed the anti‐ferroptosis effects of r‐Metrnl after SAH. (a) Representative bands of western blot. (b–i) Quantitative analysis of p‐C‐KIT, C‐KIT, p‐AMPK, AMPK, Nrf2, SLC7A11, GPX4, FSP1, FTH, and ACSL4 expression in the ipsilateral hemisphere at 24 h after SAH. *n* = 6 per group. (j, k) ELISA analysis of MDA and GSH levels. *n* = 6 per group. (l, m) Representative image and quantitative analyses of 4HNE at 24 h after SAH. Scale bar = 100 μm, *n* = 4 per group. Data are represented with mean ± SD. *p < 0.05 vs. Sham, ^#^ *p* < 0.05 vs. SAH + PBS, ^@^ *p* < 0.05 vs. SAH + r‐Metrnl + DMSO (i.c.v.).

To investigate the neuroprotective mechanism of r‐Metrnl, we examined the involvement of the C‐KIT receptor tyrosine kinase and AMPK pathways. The C‐KIT inhibitor ISCK03 and the AMPK inhibitor dorsomorphin were used. As shown in Figure 8, p‐C‐KIT, p‐AMPK, Nrf2, ACSL4, and MDA levels were significantly increased, and the expression of SLC7A11, GPX4, FSP1, FTH, and GSH was markedly reduced in the SAH group compared to the sham group (*p* < 0.05, Figure 8a–k). Treatment with r‐Metrnl significantly upregulated p‐C‐KIT, p‐AMPK, Nrf2, SLC7A11, GPX4, FSP1, FTH, and GSH, while decreasing ACSL4 and MDA levels compared to the SAH + PBS group at 24 h after SAH (*p* < 0.05, Figure 8a–k). However, ISCK03 administration prior to SAH induction caused a significant reduction in p‐C‐KIT level compared to the SAH + r‐Metrnl + DMSO (i.c.v.) group (*p* < 0.05, Figure 8a,b). Furthermore, co‐administration of ISCK03 or dorsomorphin led to elevated ACSL4 and MDA levels and reduced p‐AMPK, Nrf2, SLC7A11, GPX4, FSP1, FTH, and GSH compared to the SAH + r‐Metrnl + DMSO (i.c.v.) group (*p* < 0.05, Figure 8a–k). Immunofluorescence analysis of 4HNE showed that the level of lipid peroxidation in the SAH + r‐Metrnl + ISCK03 group and the SAH + r‐Metrnl + dorsomorphin group was significantly higher than that in the SAH + r‐Metrnl + DMSO (i.c.v.) group (*p* < 0.05, Figure 8l,m).

**FIGURE 9:**
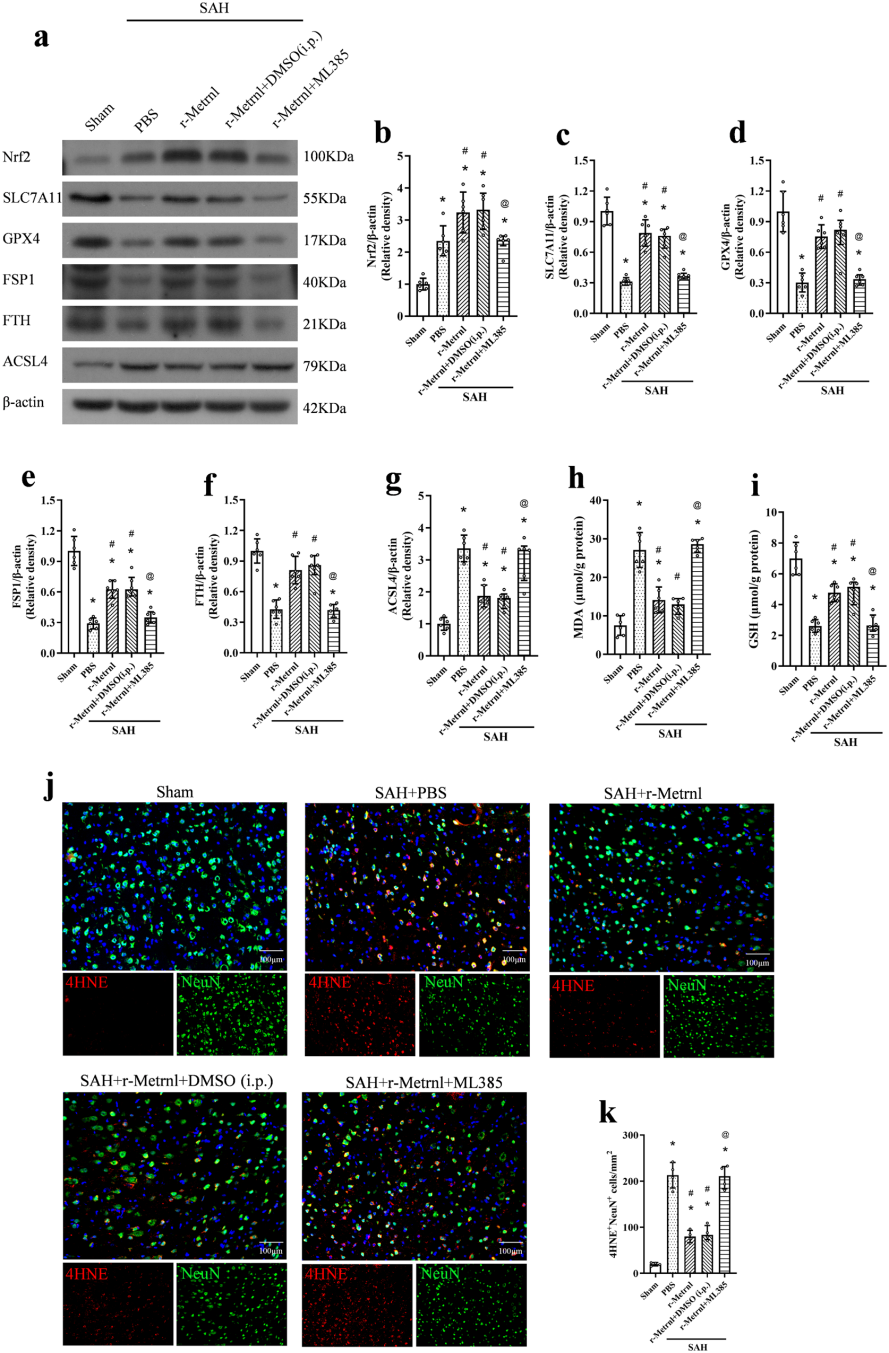
Inhibition of Nrf2 reversed the anti‐ferroptosis effects of r‐Metrnl after SAH. (a) Representative bands of western blot. (b–i) Quantitative analysis of Nrf2, SLC7A11, GPX4, FSP1, FTH, and ACSL4 expression in the ipsilateral hemisphere at 24 h after SAH. *n* = 6 per group. (h, i) ELISA analysis of MDA and GSH levels. *n* = 6 per group. (j, k) Representative image and quantitative analyses of 4HNE at 24 h after SAH. Scale bar = 100 μm, *n* = 4 per group. Data are represented as mean ± SD. *p < 0.05 vs. Sham, ^#^ *p* < 0.05 vs. SAH + PBS, ^@^ *p* < 0.05 vs. SAH + r‐Metrnl + DMSO (i.p.).

To further explore downstream molecules in this pathway, we used the selective Nrf2 inhibitor ML385 that was administered intraperitoneally 1 h before SAH induction. ML385 significantly reduced the expression of Nrf2, SLC7A11, GPX4, FSP1, FTH, and GSH, and increased ACSL4 and MDA levels 24 h after SAH (*p* < 0.05, Figure 9a–i). In addition, the level of lipid peroxidation of the SAH + r‐Metrnl + ML385 group was significantly higher than that of the SAH + r‐Metrnl + DMSO (i.p.) group (*p* < 0.05, Figure 9j,k).

### 3.8 R‐Metrnl Inhibits Ferroptosis via C‐KIT/AMPK/Nrf2 Signaling Pathway in Hb‐Stimulated Primary Neurons

**FIGURE 10:**
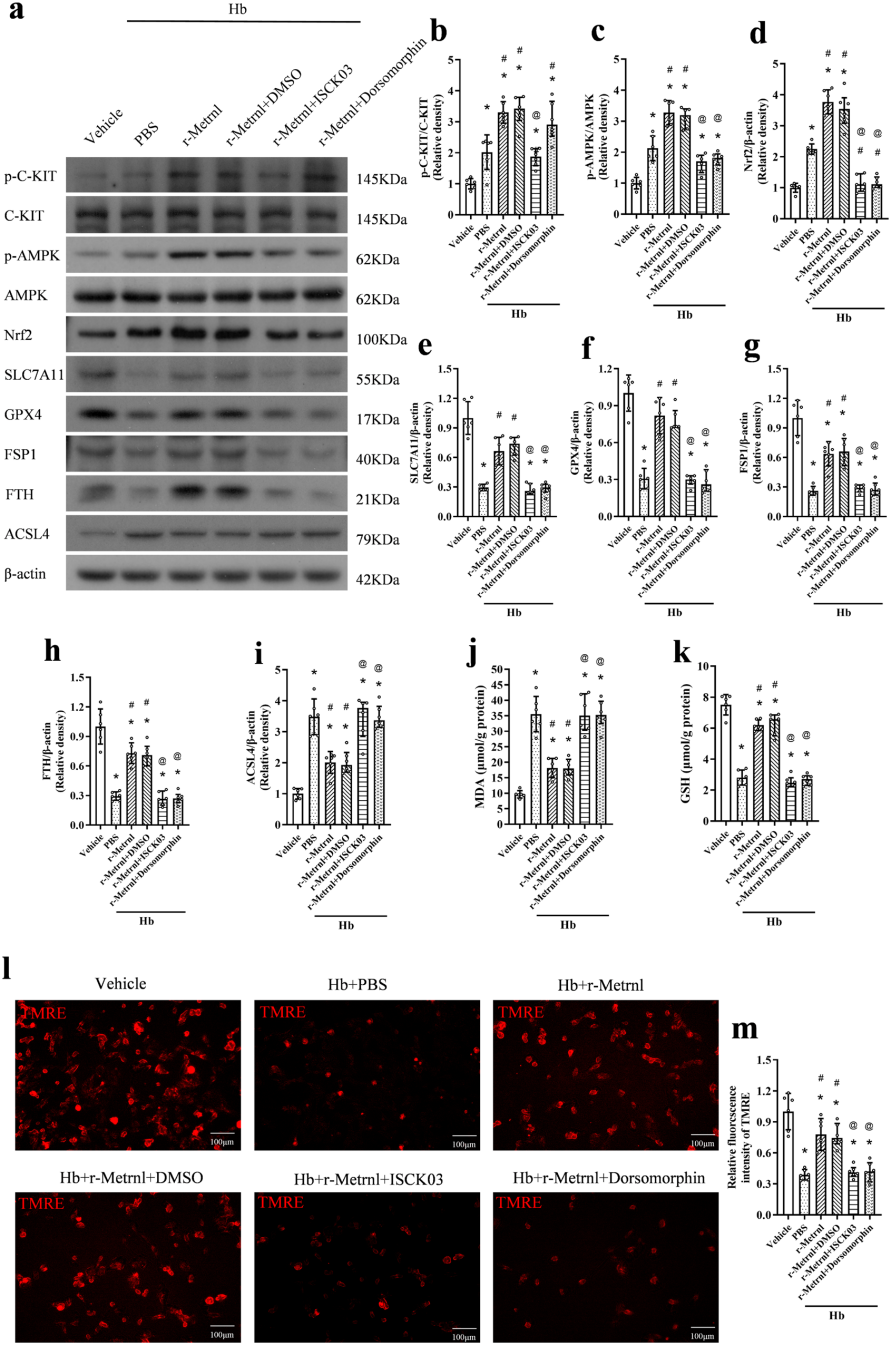
Inhibition of C‐KIT or AMPK reversed the anti‐ferroptosis effects of r‐Metrnl in Hb‐stimulated primary neurons. (a) Representative bands of western blot. (b–i) Quantitative analysis of p‐C‐KIT, C‐KIT, p‐AMPK, AMPK, Nrf2, SLC7A11, GPX4, FSP1, FTH, and ACSL4 expression in the ipsilateral hemisphere at 24 h after SAH. *n* = 6 per group. (j, k) ELISA analysis of MDA and GSH levels. *n* = 6 per group. (l, m) Representative image and quantitative analyses of TMRE. Scale bar = 100 μm, *n* = 6 per group. Data are represented with mean ± SD. *p < 0.05 vs. Vehicle, ^#^ *p* < 0.05 vs. Hb + PBS, ^@^ *p* < 0.05 vs. Hb + r‐Metrnl + DMSO.

Primary neuron culture experiment was performed to verify whether the effects of Metrnl were mediated by attenuating neuronal ferroptosis through the C‐KIT/AMPK/Nrf2 signaling pathway. Consistent with the results in vivo, Hb stimulation significantly increased p‐C‐KIT, p‐AMPK, Nrf2, ACSL4, and MDA levels, and the expression of SLC7A11, GPX4, FSP1, FTH, and GSH was markedly reduced (*p* < 0.05, Figure 10a–k). Compared to the Hb + PBS group, the expression of p‐C‐KIT, p‐AMPK, Nrf2, SLC7A11, GPX4, FSP1, FTH, and GSH increased in the Hb + r‐Metrnl treated group (*p* < 0.05, Figure 10a–k). Meanwhile, the expression of ACSL4 and MDA decreased (*p* < 0.05, Figure 10a–k). ISCK03 treatment significantly decreased p‐C‐KIT expression compared to the Hb + r‐Metrnl + DMSO group. Administration of ISCK03 or dorsomorphin reversed the effects of r‐Metrnl on p‐AMPK, Nrf2, SLC7A11, GPX4, FSP1, FTH, ACSL4, MDA, and GSH expression (*p* < 0.05, Figure 10a–k).

Meanwhile, the TMRE probe test for mitochondrial membrane potential consistently showed that decreased mitochondrial membrane potential after Hb stimulation was restored by r‐Metrnl administration. These effects were reversed by the inhibition of C‐KIT or AMPK (*p* < 0.05, Figure 10l,m).

## 4 Discussion

This study is the first to investigate the impact of r‐Metrnl on neuronal ferroptosis in a rat model of SAH and to explore the underlying molecular mechanisms. The key novel findings include the following. (1) The expression levels of endogenous Metrnl and its receptor C‐KIT significantly increased, peaking at 24 h after SAH. Both Metrnl and C‐KIT were co‐localized with neurons. (2) Knockdown of endogenous Metrnl using shRNA significantly worsened neurological deficits and neuronal ferroptosis 24 h after SAH in rats. (3) Exogenous r‐Metrnl treatment effectively reduced neurobehavioral deficits, neuronal degeneration, and ferroptosis. (4) Exogenous r‐Metrnl treatment also reduced neuronal damage in the ipsilateral hippocampus 28 days after SAH. (5) Inhibition of the C‐KIT/AMPK/Nrf2 pathway reversed the neuroprotective effects of r‐Metrnl on lipid peroxidation and neuronal ferroptosis both in vivo and in vitro. These results indicated that r‐Metrnl protected against ferroptosis primarily through the C‐KIT/AMPK/Nrf2 signaling pathway after SAH.

Metrnl, a novel secreted protein consisting of 311 amino acids, is found in various tissues, including the liver, skeletal muscle, heart, brain, and adipose tissues [32, 33]. Recent research has examined Metrnl expression in the hippocampus, cerebellum, and cortex [34]. It is noted that Metrnl expression increases in various inflammatory conditions [14, 15]. Consistent with these findings, our study indicated an elevated expression of Metrnl in SAH, characterized by an excessive inflammatory response. Additionally, we observed that Metrnl is expressed in neurons within the ipsilateral hemisphere. A recent study by Reboll MR first identified Metrnl as a high‐affinity ligand for the C‐KIT receptor tyrosine kinase [15]. C‐KIT is widely distributed in the central nervous system and is crucial for neural cell development and homeostasis [35]. Previous studies have revealed that C‐KIT is expressed in neurons, microglia, and other immune cells [36, 37]. Similarly, double immunofluorescence in our study confirmed that C‐KIT is abundantly expressed in neurons following SAH. Additionally, it has been reported that phosphorylation of C‐KIT significantly increased following brain injuries in experimental traumatic brain injury [38]. Similarly, our findings indicate that p‐C‐KIT expression substantially increased after SAH, reaching a peak at 24 h after SAH.

Ferroptosis is identified by several biochemical characteristics: iron accumulation, antioxidant function reduction, lipid peroxidation, and escalated ROS generation [39]. Regarding morphology, ferroptosis is different from other types of cell demise, primarily showing significant shrinkage of mitochondria, elevated membrane density, and a reduction or complete absence of mitochondrial cristae [40]. SLC7A11, a sodium‐independent cystine–glutamate antiporter also known as xCT, is crucial in generating intracellular GSH against oxidative stress. When SLC7A11 is inhibited, GSH levels drop and GPX4 activity is impaired, leading to the iron‐dependent accumulation of ROS [39]. The escalation of ROS leads to interactions with polyunsaturated fatty acids (PUFAs) within lipid membranes, thereby initiating lipid peroxidation [41]. Proteins associated with lipid metabolism, such as ACSL4 and ALOX15, are instrumental in ferroptosis. ACSL4's function is to transform PUFAs into fatty PUFA–CoA, which is essential for producing and modifying PUFA‐phosphatidylethanol amines (PEs) [40]. Oxidation of specific PEs, particularly those rich in arachidonic or adrenic acid, is a key process that propels cells toward ferroptosis [42]. Iron accumulation is a key characteristic of ferroptosis. Ferritin is a cytosolic iron storage protein that proves to be crucial for maintaining cellular iron metabolism balance [43]. FTH is a component of the heavy subunit in ferritin and functions as ferroxidase, which is essential for converting Fe^2+^ into Fe^3+^ for safe iron sequestration within ferritin nanocages. This conversion helps mitigate iron overload and limits the availability of Fe^2+^ for ROS generation [44]. Additionally, research discovered that FSP1 contributed to the restoration of COQ10 through a process involving NAD(P)H. This FSP1–COQ10–NAD(P)H pathway operated independently of GPX4 and GSH, which was crucial in preventing lipid peroxidation associated with ferroptosis [45]. Several studies suggested that ferroptosis plays a significant role in neuronal damage following both ischemic and hemorrhagic strokes, especially in the case of SAH, which is known for its high fatality and morbidity rates [46]. Hb degradation leads to an excessive liberation of Fe^2+^, which disrupts mitochondrial function and consequently promotes the accumulation of lipid‐derived radicals and peroxides, ultimately inducing neuronal ferroptosis [2]. Our study observed significant alterations in the expression of GSH, FTH1, SLC7A11, GPX4, FSP1, and ACSL4, and lipid peroxide accumulation after SAH. These findings indicate that ferroptosis is a key point in brain injury following SAH. Consequently, suppressing neuronal ferroptosis may offer a promising approach to mitigate brain damage caused by SAH.

Metrnl has shown potential in mitigating oxidative stress and ferroptosis. Intravenous administration of Metrnl decreased ROS levels in macrophages and inhibited the phenotypic differentiation of iNOS macrophages in a cecal ligation puncture model [47]. Exogenous Metrnl also significantly reduced ROS generation in IL‐1β–treated chondrocytes [48]. Considering that lipid peroxidation products, ROS derived from lipids, and mitochondrial damage are characteristic indicators of ferroptosis [49], we speculated that r‐Metrnl treatment might suppress neuronal ferroptosis following SAH. Our findings indicate that Metrnl treatment upregulates the expression of SLC7A11, FSP1, FTH, and GPX4. Additionally, following r‐Metrnl administration, a reduction in ACSL4 expression, an enzyme that facilitates lipid peroxidation, was observed in the brains of SAH rats. Simultaneously, r‐Metrnl treatment decreased the MDA level and the number of 4‐HNE–positive neurons and mitigated mitochondrial damage. Conversely, Metrnl knockdown exacerbated lipid peroxidation and neuronal damage. Our study demonstrates that the exogenous delivery of r‐Metrnl effectively suppresses neuronal ferroptosis following SAH induction.

Accumulating evidence suggests that the activation of AMPK plays a key role in most of the biological effects of Metrnl [33, 50]. Current investigations have highlighted the protective role of AMPK in ferroptosis. Energy stress activates AMPK and AMPK, then phosphorylates and inactivates acetyl‐CoA carboxylase, resulting in restrained biosynthesis of PUFAs and other fatty acids and ferroptosis inhibition [51]. A recent study showed that Metrnl enhanced the repair of myocardial infarcts through binding to the KIT receptor tyrosine kinase in mice [15]. Previous research has shown that C‐KIT activation promotes cell survival [12]. In our current SAH study, we utilized C‐KIT and AMPK inhibitors to reverse the neuroprotective effects of Metrnl, thereby validating the significance of C‐KIT/AMPK signaling in Metrnl's neuroprotection against ferroptosis after SAH and Hb stimulation.

Nrf2 is essential in maintaining cellular redox balance and resisting ferroptosis by promoting the transcription of antioxidant genes [52]. Most genes associated with ferroptosis, such as ferritin, ferroportin, GPX4, and SLC7A11, are regulated by Nrf2 [52]. Metrnl overexpression has been shown to improve Nrf2 protein levels and nuclear accumulation in hearts with doxorubicin treatment, reducing ROS levels [11]. Furthermore, the activation of KIT signaling mitigates light‐induced degeneration of photoreceptors by promoting the nuclear translocation of the transcription factor NRF2 and enhancing the expression of the antioxidant gene Hmox1 [53]. Moreover, studies have revealed that Nrf2, a major downstream mediator of AMPK, is essential for AMPK‐induced prevention of ferroptosis [23, 54]. Although the role of Nrf2 in r‐Metrnl's anti‐ferroptosis effect is not fully understood, our study showed that r‐Metrnl increases Nrf2 expression. When Nrf2 was blocked with ML385, the neuroprotective effect of r‐Metrnl and its induction of Nrf2 were abolished. These findings substantiate that r‐Metrnl regulates SLC7A11 and GPX4 levels via the C‐KIT/AMPK/Nrf2 signaling pathway, crucial in the prevention of ferroptosis.

There are several limitations to this study. First, we used Metrnl knockdown shRNA for the genetic knockdown of brain Metrnl. Employing a transgenic animal model for further validation is essential to substantiate our findings. Second, our research focused on the C‐KIT/AMPK/Nrf2 pathway as the key mechanism behind r‐Metrnl's neuroprotective effects after SAH. However, other molecular pathways that may contribute to r‐Metrnl's benefits should not be ruled out, and additional studies are required to explore these possibilities and underlying mechanisms. Lastly, we did not evaluate gender differences in the current study.

**FIGURE 11:**
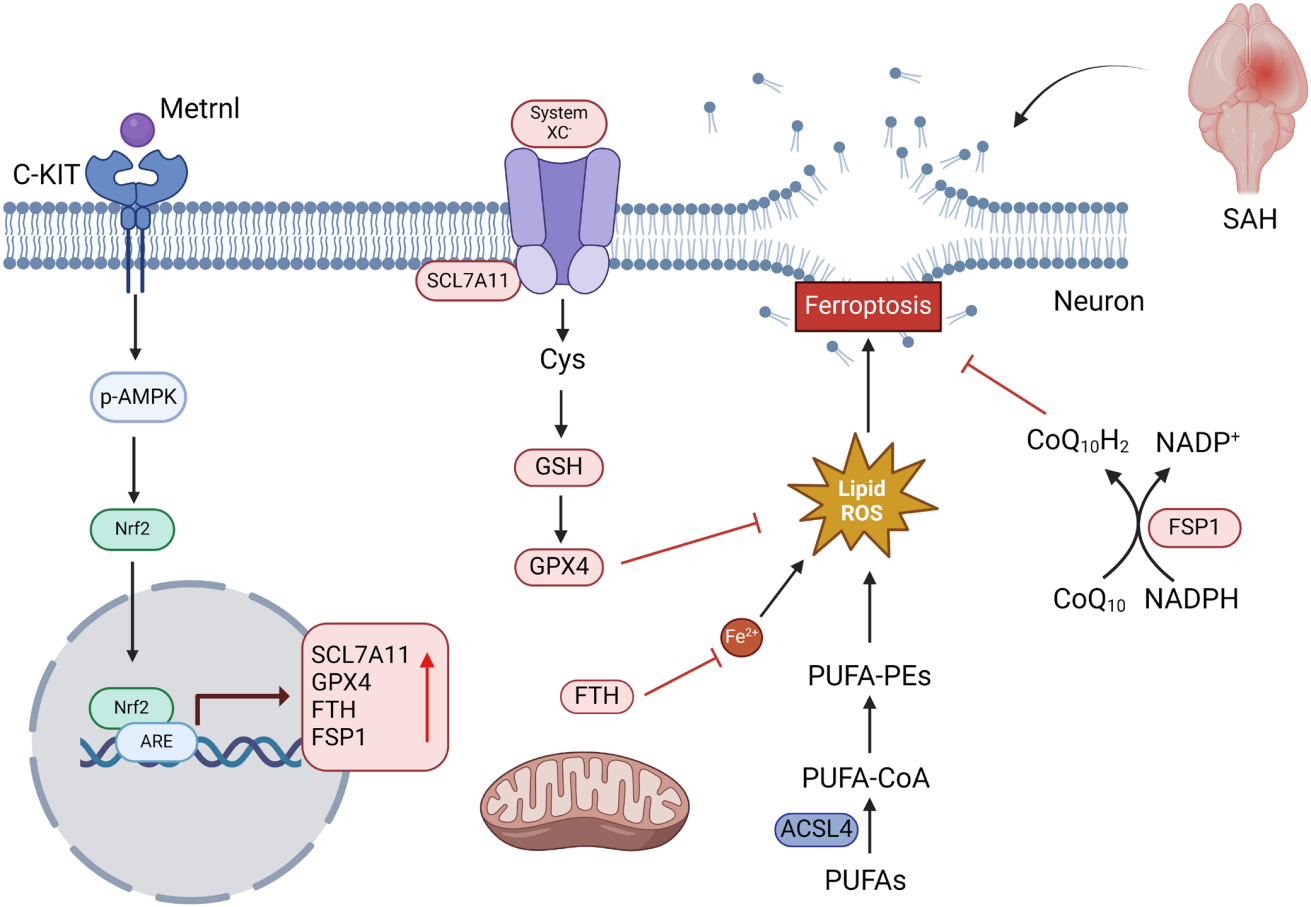
Metrnl attenuated neuronal ferroptosis via the activation of C‐KIT/AMPK/Nrf2 signaling pathway after SAH.

Overall, our study demonstrated that r‐Metrnl protects against SAH‐induced neurological damage by inhibiting ferroptosis. The therapeutic effect of r‐Metrnl is in part realized through the engagement of the C‐KIT/AMPK/Nrf2 signaling axis (Figure 11). Our research may provide insights into the new injury mechanism of Metrnl/C‐KIT interaction–induced neuronal ferroptosis after SAH, suggesting Metrnl as a potential target for SAH treatment.

## Author Contributions

Q.H., Y.Z. and Q.Y. conceived the study. Q.H., Y.Z., J.L., Y.Y., H.Z., X.L., F.W., Y.C. and Y.T. performed experiments. Q.H., Q.Y. and Y.Z. wrote the manuscript. Q.H., J.Y., L.H. and L.W. collected and analyzed the data. Q.H., Y.Z., J.L. and Q.Y. reviewed and edited the manuscript. All authors have read and agreed to the published version of the manuscript.

## Conflicts of Interest

The authors declare no conflicts of interest.

## Supporting information


Appendix S1

## Data Availability

The data supporting this study's findings are available from the corresponding author upon request.
