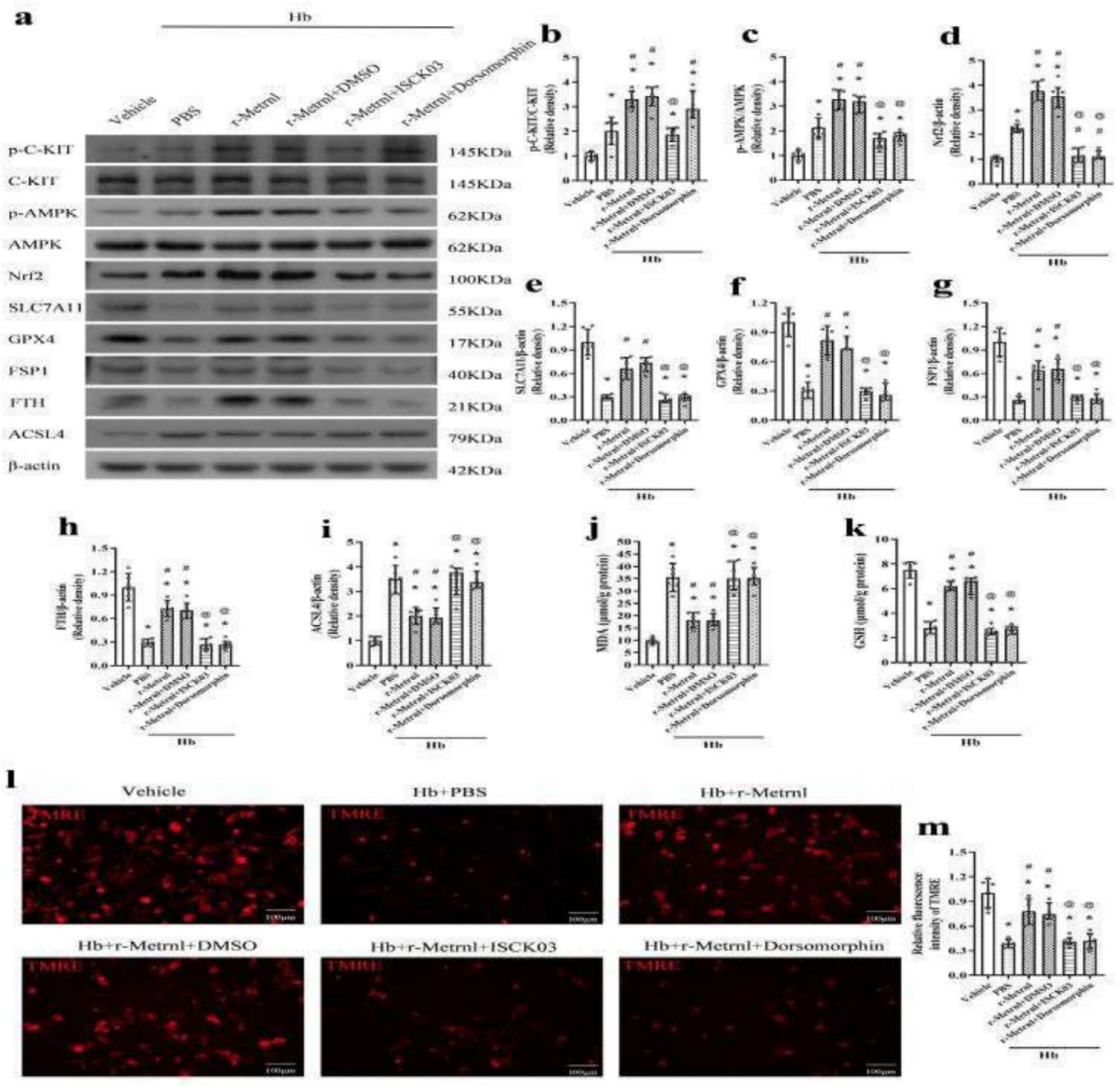# Correction to “Metrnl/C‐KIT Axis Attenuates Early Brain Injury Following Subarachnoid Hemorrhage by Inhibiting Neuronal Ferroptosis”

**DOI:** 10.1111/cns.70494

**Published:** 2025-06-23

Zhou, Y., Li, J., Yuan, Y., Zhang, H., et al. (2025), Metrnl/C‐KIT Axis Attenuates Early Brain Injury Following Subarachnoid Hemorrhage by Inhibiting Neuronal Ferroptosis. *CNS Neuroscience & Therapeutics*, 31: e70286. https://doi.org/10.1111/cns.70286

In the original version of this article, there was an error in Figure 10L. Specifically, the TMRE representative image in Hb + r‐Metrnl + ISCK03 group was incorrect. The correct image is provided below. The correction does not affect the results and conclusions.

We apologize for this error in the article.