# Supplementary figures and images for: Metrnl/C‐KIT Axis Attenuates Early Brain Injury Following Subarachnoid Hemorrhage by Inhibiting Neuronal Ferroptosis
Source: CNS Neurosci Ther. 2025 Feb 21;31(2):e70286. doi: 10.1111/cns.70286 (PMC11843251; doi:10.1111/cns.70286)

Figure 2

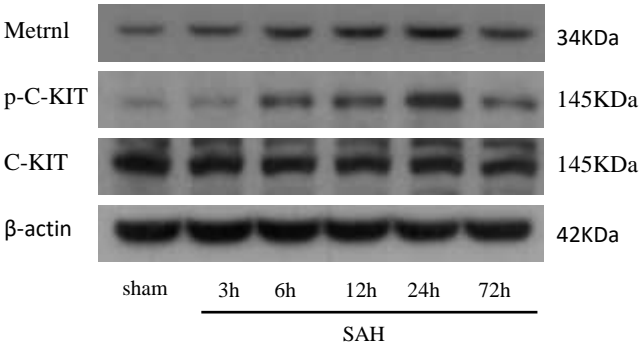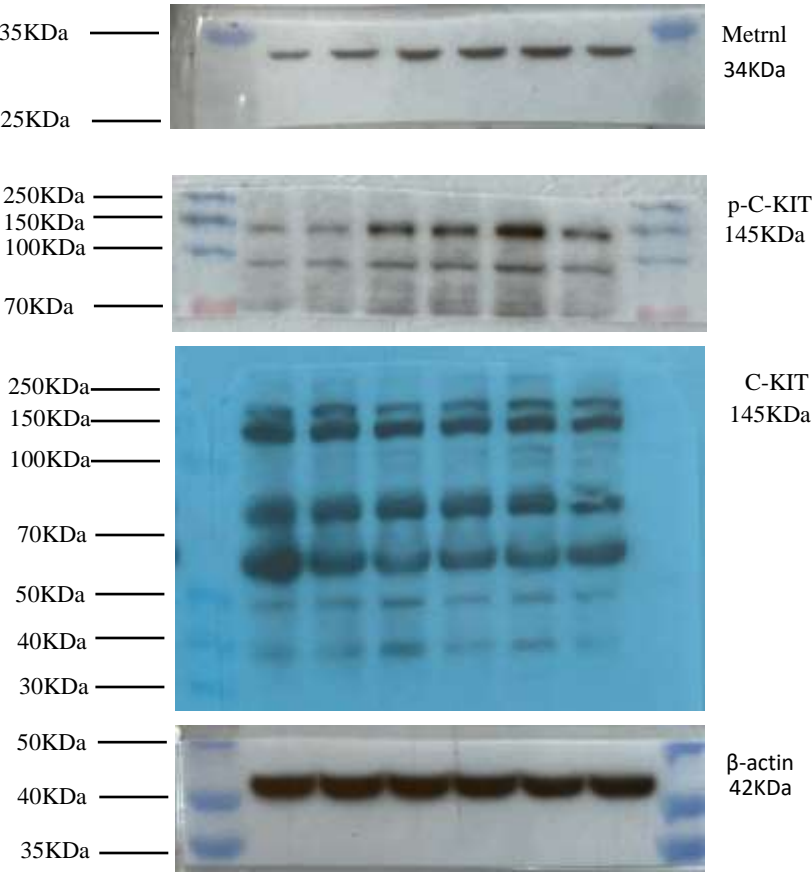

Figure 3

**a**

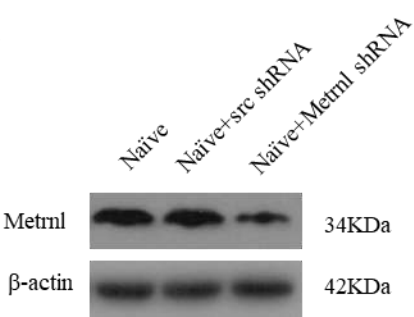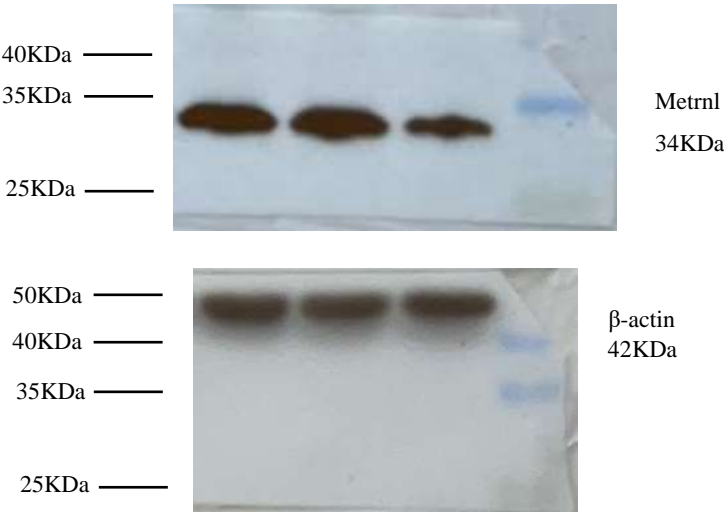

Figure 4

**a**

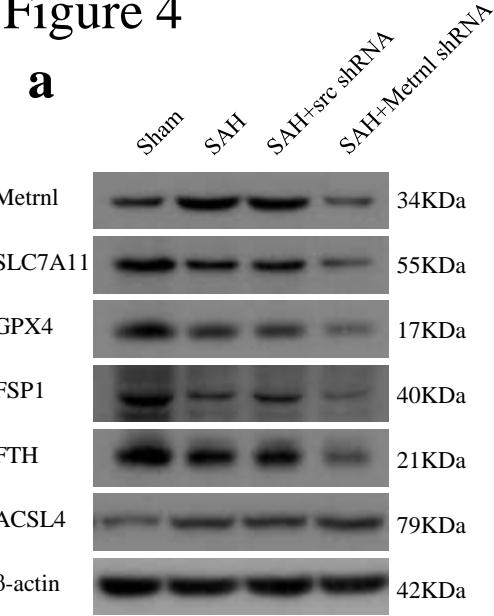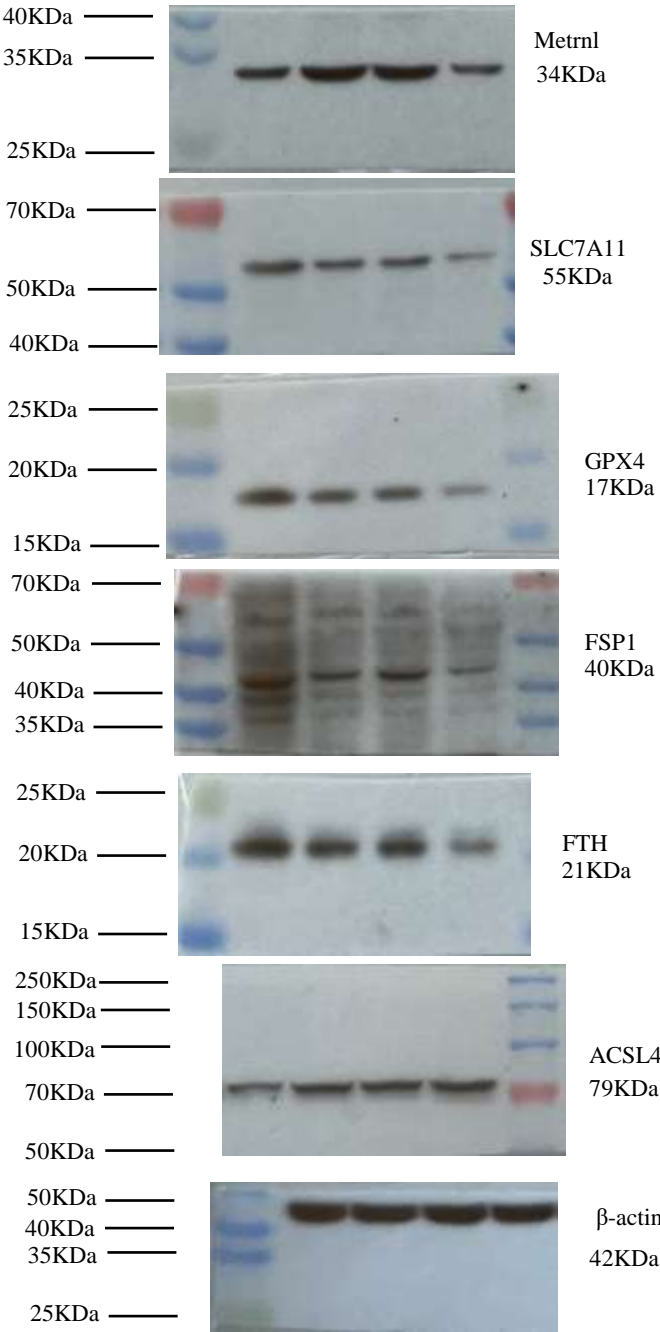

Figure 6

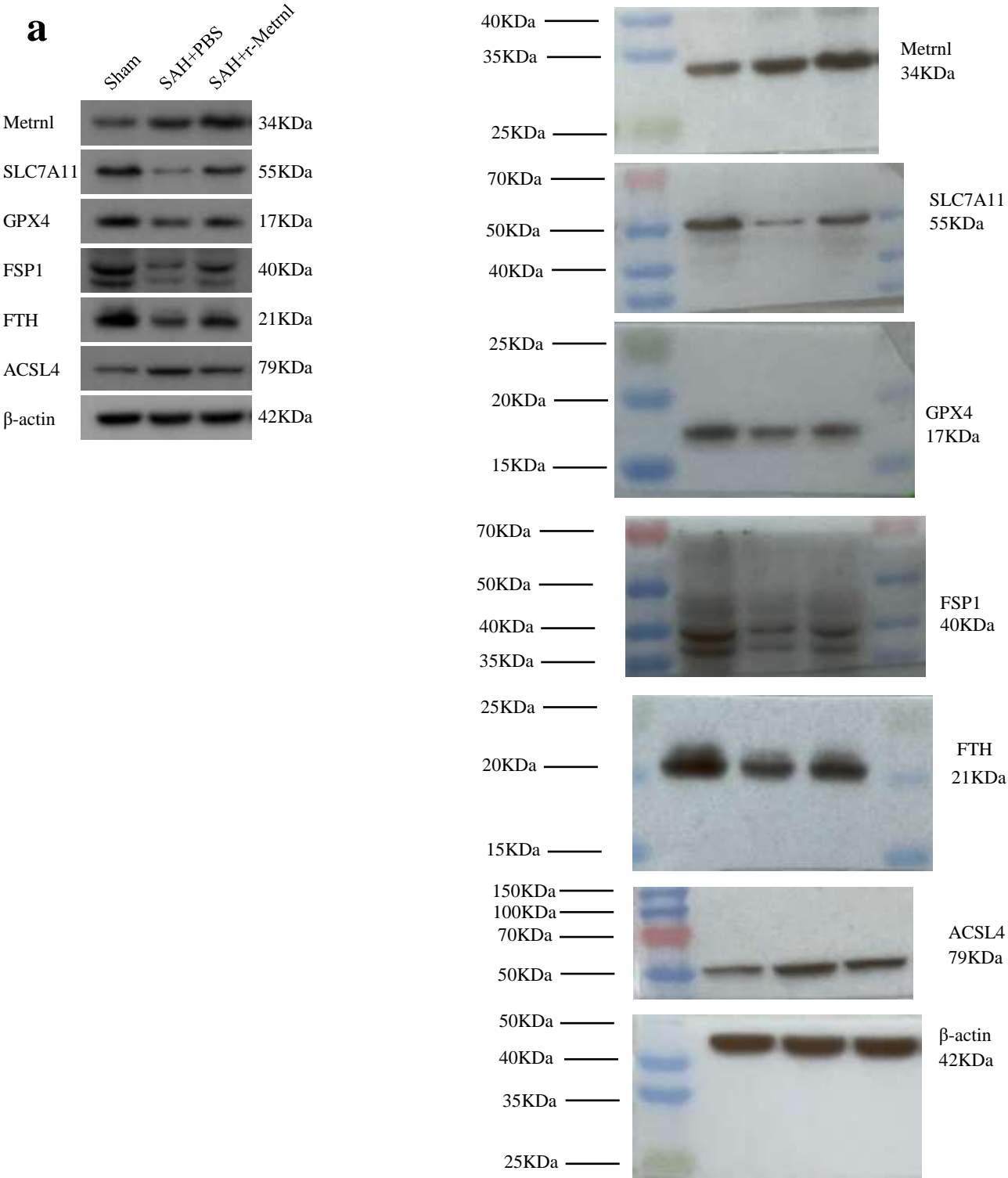

Figure 8

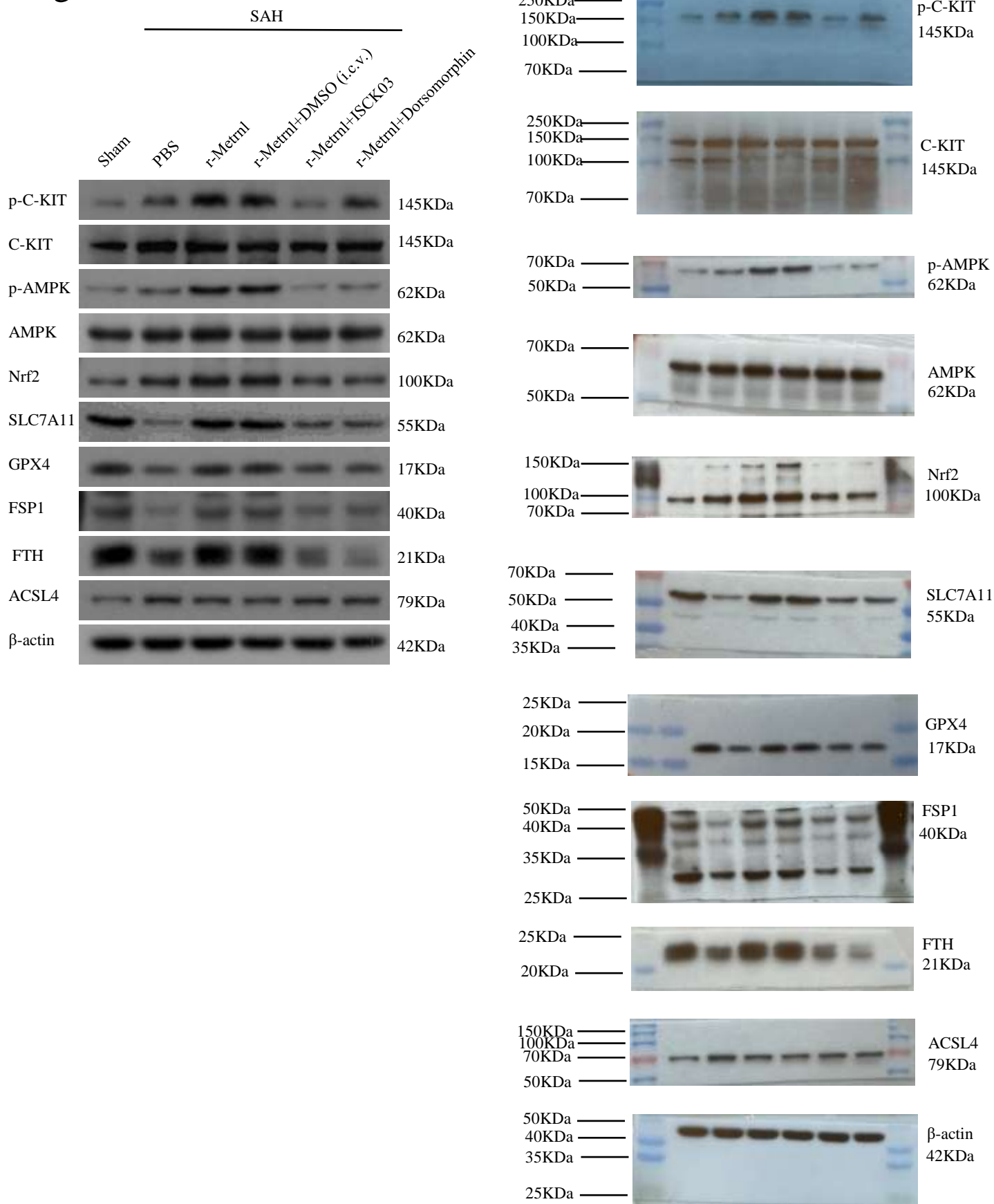

Figure 9

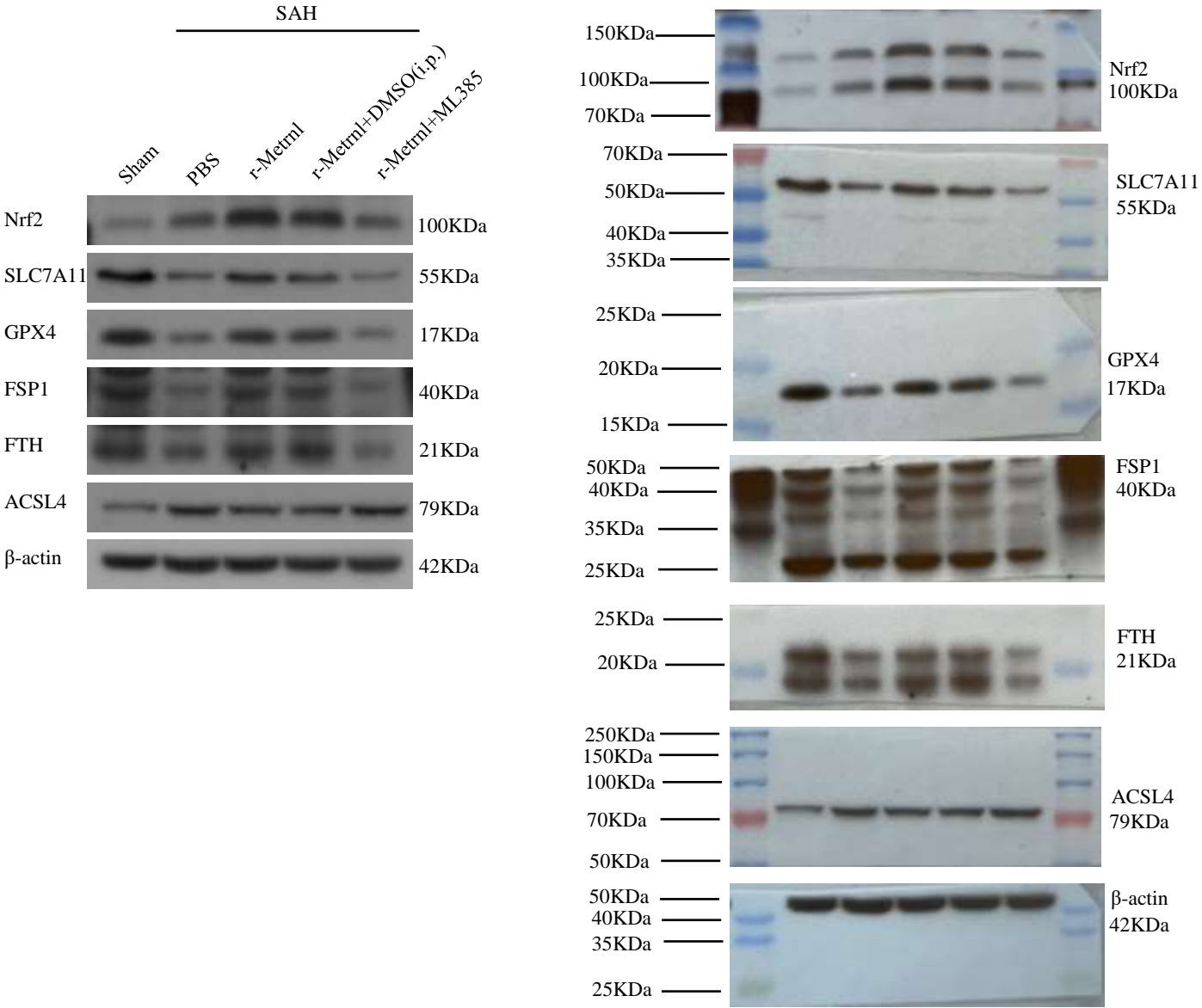

Figure 10

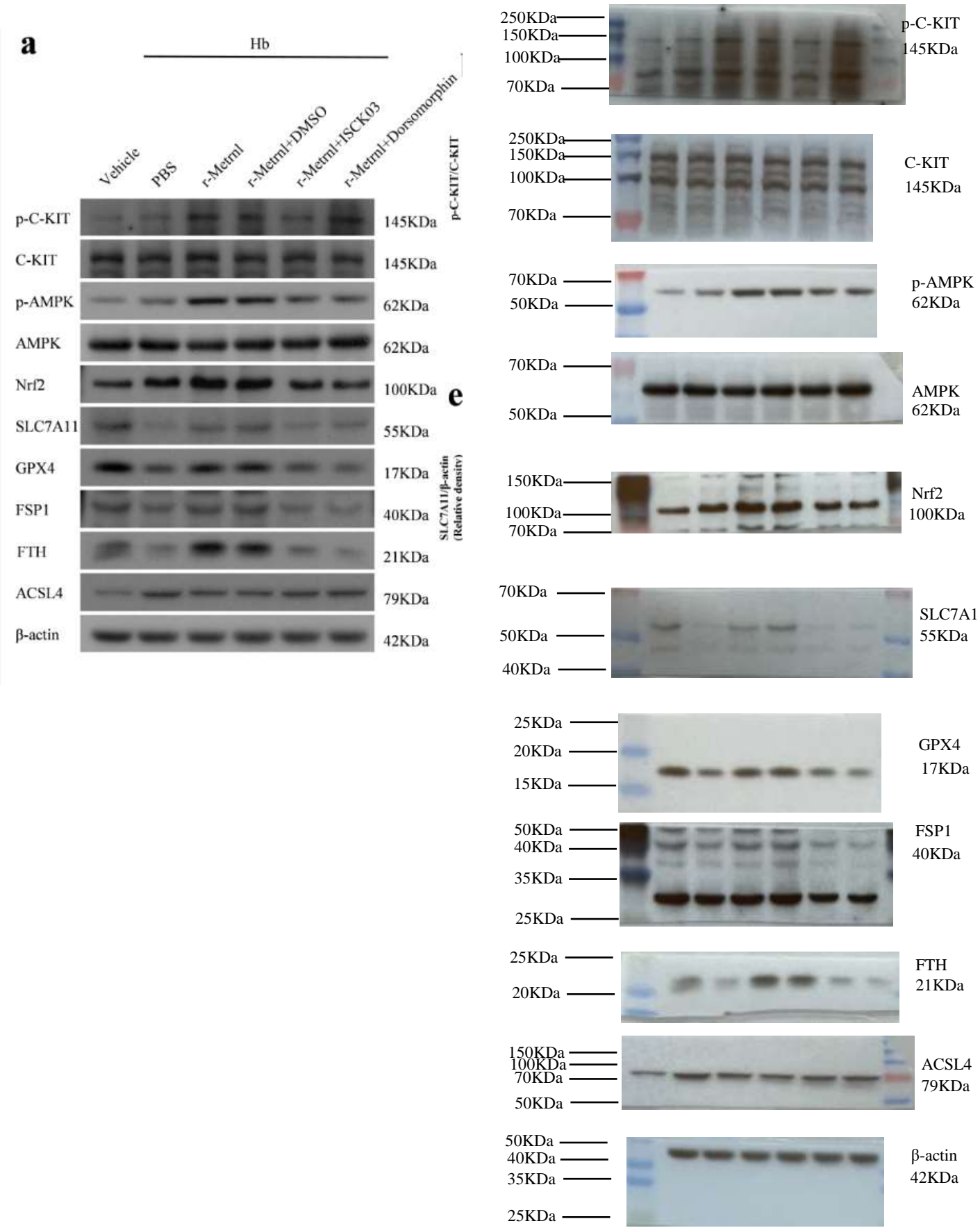

Supplement: Supplementary file 1 — Appendix S1 [file CNS-31-e70286-s001.pdf]
